# Smart biomaterials for skeletal aging repair and regeneration

**DOI:** 10.1038/s41413-026-00505-9

**Published:** 2026-02-14

**Authors:** Dingfa Liang, Hufei Wang, Yu Jiang, Zeyuan Zhang, Tianjunke Zhou, Siliang Ge, Shuhuai Tan, Kaihua Qin, Yilin Wang, Xisheng Lin, Yong Xie, Houchen Lyu, Licheng Zhang

**Affiliations:** 1 Senior Department of Orthopedics, the Fourth Medical Center of Chinese PLA General Hospital, Beijing, China; 2 National Clinical Research Center for Orthopedics, Sports Medicine and Rehabilitation, Beijing, China; 3 Medical School of Chinese PLA, Beijing, China; 4 Department of Rehabilitation, the Second Medical Center of Chinese PLA General Hospital, Beijing, China; 5 Senior Department of Orthopedics, the Sixth Medical Center of Chinese PLA General Hospital, Beijing, China

**Keywords:** Pathogenesis, Bone

## Abstract

Skeletal aging associated with diverse age-related disorders is increasing due to unhealthy diets, stressful lifestyles, and rapid aging. Repair and regeneration of aging skeletons are a global issue. Despite the self-healing ability of bone and the availability of various treatment strategies, degenerative bone repair and regeneration face significant problems due to unbalanced bone remodeling and a lack of active treatment strategies. The development of smart materials has created opportunities for degenerative bone repair and regeneration. The smart materials are responsive to endogenous/exogenous stimuli with tailored structure and function, which can promote skeletal aging repair and regeneration. Thus, in this study, skeletal aging is recognized as the progressive state that begins from peak bone mass to pathophysiological state and disorder conditions. We have introduced and characterized skeletal aging from the perspectives of cell-matrix-microenvironment and macrostructure-function-mechanical properties, for which systemic smart drug delivery systems and local smart scaffolds are designed. The smart drug delivery systems undergo conformation change and phase transition upon stimuli to release drugs at time- and site-specific to promote aging bone repair. Smart scaffolds with versatility and mechanical strength can replace bone defects to provide a tissue repair and regeneration microenvironment. Endogenous disease microenvironments and/or external physical triggers stimulate scaffold activation, which release bioactive factors to accelerate bone regeneration. This manuscript discusses the manufacturing techniques of these smart materials and presents key challenges and future directions for clinical translation, emphasizing their potential for personalized treatment and targeted therapy of skeletal aging.

## Introduction

In the recent evolutionary past, human life expectancy and population growth have significantly increased, accompanied by various age-related disorders and chronic morbidities. These conditions include skeletal aging, neurodegenerative disorders, and cardiometabolic diseases.^1,2^ Of these, skeletal aging is a progressive status from pathophysiological degeneration with impaired bone homeostasis to pathogenic diseases with reduced bone mass and quality.^3,4^ Dysregulated biological processes, cellular senescence, cytokine disequilibrium, tissue impairment and skeletal fracture are responsible for age-related degeneration and dysfunction, resulting in degenerative intervertebral discs, degraded articular cartilage, and loss of bone.^3,5^ Only 31%–36% of people over the age of 70 have normal bones. Skeletal aging imposes an increasing disease burden, and the consequences, such as osteoporosis and osteoarthritis (OA), are deleterious to quality of life and endanger lives.^3,6^

Many clinical therapeutic strategies are applied to treat complex disorders of skeletal aging, such as palliative medication and regenerative approaches. Current bone-modifying agents, such as bisphosphonates, estrogens, and denosumab, can restore bone mineral and bone mass, thereby partially improving or alleviating skeletal aging, especially for osteoporosis.^7^ Although the pharmacologic interventions are well-tolerated, there are many limitations and side effects for actual use due to the poor aqueous solubility, drug instability in serum, subpar circulation time (<24 h), and poor tissue localization. Less than 1% of conventional systemically administered therapies reach the bone tissue.^8^ Early drug delivery systems based on the physicochemical properties (i.e., size, surface charge, surface chemistry, hydrophobicity) and inherent biological processes (mononuclear phagocytic system, enhanced permeability and retention) achieve passive tissue localization, resulting in short-term localization and lacking of tissue-specific targeting.^9^ Regenerative therapy, including mesenchymal stem cell (MSC) transplantation, has been long examined and proposed via direct differentiation, attraction and recruitment effect, and secretory substance.^10,11^ However, the uncertain stem cell fate is the main obstacle to stem cell-based regenerative therapy since the randomly distributed transplanted MSCs and short-term engraftment.^12^ Therefore, rational design and development of novel therapeutic strategies for skeletal aging repair and regeneration are imperative in the aging society.

Smart biomaterials are the newly developed alternative approaches with instructive/inductive or triggering/stimulating effects on cells and tissues.^13^ They are considered engineered materials that are stimulated by endogenous/exogenous stimuli, with tailored structure and function for skeletal aging repair and regeneration.^3,5^ These pathological alterations of skeletal aging serve as internal stimuli that can recruit time-controlled and site-specific bioactive factors (drug or bone-favour cytokines) released from smart biomaterials. Besides, exogenous stimuli (e.g., light, ultrasound, electrical signal, magnetic and mechanical force) can trigger the automated release from smart biomaterials.^14^ Sophisticated versions of bioresponsive biomaterials demonstrate significantly improved release efficiency and provided tailored delivery of treatments to certain cells, receptors, or biological processes. A novel biocompatible MMP-13/pH-responsive ferritin nanocages (CMFn) loaded with hydroxychloroquine (CMFn@HCQ) was created for cartilage-targeting imaging and therapy. The cumulative release of hydroxychloroquine in CMFn@HCQ was improved from ~36% to ~83% upon the dual MMP-13 and pH stimuli.^15^ Also, smart biomaterials can provide physiochemical signaling components (like mechanical signaling and controllable micromotion) and simulate the biological architectures and functionalities of defective bone, guiding the cell behavior and fate for repair and regeneration.^16,17^ Despite this, most of the smart biomaterial systems suffer from single-stimulus dependency and limited targeted sensitivity, which affects durability and clinical adaptability of these platforms.

**Fig. 1:**
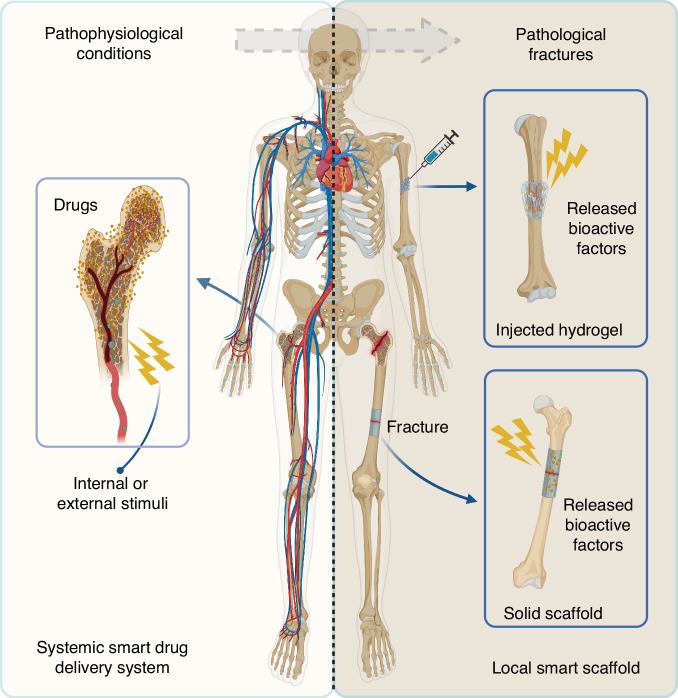
Schematic illustration of smart biomaterials for skeletal aging therapy, including a systemic smart drug delivery system and a local smart scaffold. The systemic smart drug delivery system patrolled in circulation is activated by internal or external stimuli to release drugs at the pathophysiological site to improve bone strength. The local smart scaffolds provide mechanical strength and release bioactive factors at pathological defects in response to internal or external stimuli to promote bone regeneration. Created by BioRender software (biorender.com)

In this review, we presented a systemic overview of smart biomaterials for skeletal aging therapy, especially in systemic and local strategies (Fig. 1). Various internal and external response mechanisms of smart biomaterials were included, highlighting the advanced function of multistimuli-responsive systems, which dynamically adapt to bone microenvironmental cues through synergistic effects. Furthermore, hybrid additive manufacturing and AI-driven therapeutic frameworks are presented as transformative technologies enabling patient-specific biomaterial design and accelerating clinical translation of these advanced technologies.

## Skeletal aging: from pathophysiologic conditions to pathological disorders

Natural aging is a state of holistic, progressive, and functional decline in different dimensions of the entire body, including cells, matrix, and microenvironment, from tissue structure alterations to performance changes and functional decline. Of these, aging contributes to bone degeneration and leads to age-related bone dysfunction.^18^ Skeletal aging is a progressive state of skeletal loss and dysfunction starting from the achievement of peak bone mass, which ranges from pathophysiologic conditions with increased fragility to pathogenic disorders, including osteoporosis, osteoarthritis, and pathological fracture.^18,19^ About 6% of men and 18% of women suffer from hip fractures globally, combined with increasing morbidity and mortality.^20,21^ Fractures affected by skeletal aging cause pain, impaired mobility, psychosocial distress, and loss of independence.^22^

Current clinical treatment strategies focus on palliative drug therapy and surgical regenerative therapy. Drugs used to treat skeletal aging primarily inhibit bone resorption and/or promote bone formation. But these drugs have several side effects, including osteonecrosis of the jaws, deep vein thrombosis, and an increased risk of cancer.^23–26^ Surgery is the main curative option for pathological fractures. However, a combination of surgical and biological variables can result in infection, non-union and poor prognosis.^27^ The MSCs-based regenerative strategies are considered a promising means in tissue engineering because they can stimulate osteogenesis required for bone regeneration.^28^ Compared with controls, the distal femurs of ovariectomized (OVX) rabbits treated by MSCs demonstrated an increase in trabecular thickness, bone apposition, and bone stiffness.^29^ However, numerous restrictions related to the biology of MSCs and administered methods limit their clinical applications and popularization. The risk of tumorigenesis after stem transplantation is associated with genetic instability and chromosomal aberrations of MSCs, growth regulators expressed by recipient tissue, and donor age.^30–32^ The therapeutic effectiveness of MSCs is controversial. The cell losses for their reduced stemness, short-lived viability, and random distribution contribute to low or no therapeutic effects.^33–35^ Repeated administration of MSCs results in the production of allo-antibodies, which can induce an immune response and cause inconclusive clinical benefits.^36^

### Pathophysiologic conditions of skeletal aging

**Fig. 2:**
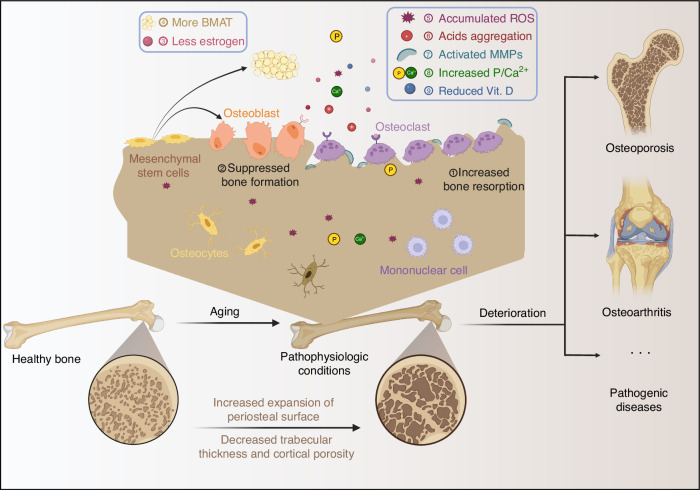
The progressive state of skeletal aging from healthy bones to pathogenic bone diseases. After the attainment of peak bone mass, bone begins to degenerate into pathophysiologic conditions with increased fragility and deteriorate into pathogenic diseases such as osteoporosis and osteoarthritis. Under the pathophysiologic conditions, the bone microenvironment undergoes a series of characteristic alterations, including an unbalanced remodeling process with increased osteoclast activity, accumulation of ROS, reduced vitamin D, reduced estrogen, and elevated bone marrow adipose tissue, which can be the targets and stimuli of smart materials. Additionally, age-related changes such as increased periosteal surface and reduced trabecular thickness and increased cortical porosity could be observed at the tissue level. Created by BioRender software (biorender.com)

The pathophysiologic progression of skeletal aging is complex, which contributes to skeletal fragility and elevated risk of pathological fracture. Herein, we addressed skeletal aging in terms of cells, matrixes, and microenvironments, following the hallmarks of aging. In addition, aging-related tissue structure alterations, organ mechanical performance changes and functional decline are recognized as the characteristics of skeletal aging that should be considered during therapeutic interventions (Fig. 2).

#### Cellular level

The MSCs within the aging bone microenvironment demonstrate deregulated gene expression, cellular senescence, and stemness depletion. The decreased expression levels of osteogenic genes, such as *osteocalcin and Runx2*, along with increased expression levels of adipocyte-specific genes including *adipocyte fatty acid-binding protein* and *peroxisome proliferator-activated receptor γ* (*PPAR-γ)* in the MSCs cause reduced bone production and increased bone marrow adipose tissue (BMAT).^37,38^ Aged MSCs exhibit higher levels of cellular senescence marked by elevated expression of p21, p53, β-galactosidase, and senescence-associated secretory phenotype compared to young donors.^39,40^ Stemness depletion, characterized by telomere dysfunction, correlates with P53/P21 pathway activation and decreased RUNX2 expression, which suppresses the differentiation of MSCs into osteoblasts, leading to bone loss and skeletal aging.^41^

Osteoblast activity tends to be diminished, accompanied by loss of proteostasis and mitochondrial dysfunction. Heat shock protein C, a key molecular chaperone of the homeostatic network of proteins, inhibits senescent osteoblasts, resulting in reduced osteoprotegerin (OPG) synthesis and diminished migration.^42,43^ Mitochondrial dysfunction with mutated DNA accumulation causes decreased osteoblast and increased osteoclast, which impairs osteogenesis and is associated with accelerated bone loss.^44^ The reduced osteoprogenitor cells and lower levels of OPG from osteoblasts, combined with endogenous metabolic and hormonal deficiencies due to aging, activate osteoclast activity and cause overloaded bone resorption. Taken together, during skeletal aging, deregulated gene expression, stemness depletion, cellular senescence, proteostasis loss, mitochondrial dysfunction, skeletal resorption overload, and altered intercellular communication drive deviations in bone remodeling processes, thereby accelerating bone loss and precipitating pathological bone disorders or fractures.

#### Matrix level

The extracellular matrix (ECM) of bone is mainly composed of organic collagen and inorganic minerals. Bone loss with age is associated with changes in the matrix composition, which can exacerbate bone brittleness.^45^ During aging, collagen cross-links increase mediated by enzymatic and non-enzymatic means.^45^ Age-related alterations in collagen (with cross-linked products), non-collagenous matrix proteins, and minerals can impair the function of mineral hydroxyapatite (HAp) crystals, affecting bone tissue rigidity and strength.

The water of bone tissue makes up approximately 10%-20% of the cortical bone volume, but drops to 5% in older age, leading to poor mechanical performance.^46^ With the loss of bone, the unbound skeletal pore water (i.e., not integrated into mineral or collagen) increases with age.^47^ These characteristic changes inhibit the ability of bones to distort under mechanical stress, leading to bone fragility and reduced fracture threshold.

#### Microenvironmental level

During the aging process, the microenvironment undergoes a series of characteristic changes. Hormone alterations are recognized as one of the characteristic markers and function as key factors that contribute to bone aging. Low level of estrogen promotes osteoclast differentiation through RANKL-RANK interaction and the mitogen-activated protein kinase (MAPK) pathway, impeding the balance of bone homeostasis.^48,49^ Estrogen decline downregulates the Wnt/β-catenin pathway and drives the imbalance between osteogenesis and adipogenesis of BMSCs. Furthermore, increased BMAT contributes to the progressive elevation of ROS, while ROS-induced oxidative stress activates PPARγ and inhibits RUNX2, promoting adipogenesis at the expense of osteogenesis.^50,51^ The increased ROS and accumulated BMAT contribute to the chronic low-grade inflammation state of the aging bone, releasing pro-inflammatory factors such as adipokines (leptin and adiponectin) and cytokines in the microenvironment.^52,53^

In addition to the changes caused by depressed BMSC, the enhanced osteoclasts also lead to significant hallmark changes. The enhanced osteoclast creates an acidic microenvironment through the secretion of organic acids (lactic acid) and protons (H^+^) through activated chlorine ion channels and proton pumps for aging bone resorption.^54^ During the bone resorption process, osteoclast in the aging environment produces more enzymes, such as MMPs and cathepsin K (CTSK). The elevated MMPs (MMP-9 and MMP-13) and CTSK expression lead to more efficient degradation of collagen fibers in the bone matrix, resulting in more fragmented collagen.^55^ Additionally, bioelectric properties (electrical conductivity and piezoelectricity) of bone tissue, based on the mineral HAp, collagen fibers and ion balance, are destroyed by enhanced osteoclast activity.^56^

During the process of bone aging, there are several characteristics of the microenvironment, including hormone alterations, accumulation of BMAT and ROS, inflammation, and related pro-inflammatory molecular patterns. In addition, osteoclast-related changes, such as pH alteration, increased enzyme production, elevated phosphate-to-calcium ratio, and unbalanced bioelectric properties, are recognized as aging-related changes within the bone microenvironment and could be the targets of therapeutic strategies.^57^

#### Macrostructure change

The normal skeleton has about 80% cortical and 20% trabecular bone. The degeneration with aging is different from males to females and from cortical to trabecular bone. Approximately, 5%-10% cortical bone and 20%-33% trabecular bone loss are accelerated during up to about 10 years of a woman’s menopausal period. Then, the loss of trabecular and cortical bones continues for the rest of life at an equal pace. Meanwhile, males experience incremental bone loss in middle age and beyond, but the total loss of cortical and trabecular bone is less severe.^58^ Males have comparatively thicker trabeculae and experience bone shrinkage rather than loss in late life, while trabecular bone loss and increased trabecular spacing are common in older females.^59^ In terms of cortical bone loss in both sexes, there is an almost linear decline in cortical bone after middle age, resulting from a decreased volume of periosteal bone deposits and elevated endocortical resorption.^60^ The total bone area was larger in males than in females for the increased expansion of the subperiosteal surface, enlargement of the medullary cavity, more declined trabecular thickness, and cortical porosity, confirmed by high-resolution peripheral quantitative computed tomography.^61^

#### Mechanical performance

The mechanical properties of bone serve as key parameters in tissue engineering, providing a basis for more suitable material design for aging bone. The alterations of mechanical performance in bone with aging reflect the reduction of mechanical properties, consisting of elastic modulus, compressive strength, tensile strength, fracture toughness, fatigue strength, shear strength, viscoelasticity, and anisotropy. The normal elastic modulus values for cortical bones range from 17 to 20 GPa and trabecular bones range from 0.2 GPa to 2 GPa. Age-related bone loss and collagen degradation, especially in trabecular bone, reduce stiffness and disrupt structure, which leads to decreased elastic modulus.^62^ The compressive strength of bone tissue is the ability to resist deformation under compression while the tensile strength is defined by the resistance to elongated force. The normal values of compressive strength are 2–12 MPa for trabecular bone and 100–180 MPa for cortical bone. The tensile strength for cortical bone is 70–150 MPa. For the aged bone, both compressive and tensile strength decrease due to mineral density and collagen skeleton degradation. Fracture toughness is the measure of bone’s resistance to the propagation of cracks and ranges from 2 to 6 MPa/m^2^ for cortical bone.^62,63^ The inhibited bone remodeling of aging bone leads to the accumulation of microdamage, which causes decreased fatigue toughness and fatigue strength, resulting in repair failure due to fatigue. Shear strength measures the bone’s ability to resist forces that cause sliding between layers. Cortical bone has a shear strength of 50–65 MPa. However, the microstructural changes that occur in aging bones, including thinning of spongy bone and reduction in collagen content reduce shear resistance, making the bones susceptible to torsional and bending stresses.^64^ Therefore, the specific mechanical changes in aging bone tissue, such as reduced elastic modulus and fracture toughness, need to be considered when designing biomaterials to achieve a more therapeutic effect.

#### Functional decline

Bone is an active connective tissue providing structural support, promoting movement, and protecting the brain, heart and lungs. It is also considered an endocrine organ that produces hormones to regulate energy metabolism such as glucose homeostasis. During the aging process, these physiological and biochemical functions of bone deteriorate.

Compared with young adults, the bone of elderly individuals undergoes a series of alterations, including a reduction in the height of the middle of the body (spine). The loss of fluid and minerals in the vertebrae leads to thinner vertebral bodies and a more curved and compressed spinal column, which can result in osteophyte formation (bone spurs).^65^ In addition, the long bones of the legs and arms become more brittle due to increased bone resorption, while their length remains unchanged.^66^ The structural support of the body skeleton becomes more brittle, and the joints become stiffer, leading to restricted movement, increased fragility, and susceptibility to fracture.

Bones are recognized as an endocrine organ that releases several key hormones influencing various metabolic processes, including glucose regulation.^67^ During aging, the endocrine function of bone tissue changes significantly, impacting the amount and functions of released hormones (e.g., fibroblast growth factor 23 (FGF23), osteocalcin, OPG, and osteopontin). The level of FGF23 is elevated due to increased bone remodeling activity and mineral imbalance during aging, which contributes to decreased vitamin D activation, impaired glucose metabolism and insulin resistance.^68^ The heightened bone turnover and increased inflammatory signaling in the bone microenvironment cause increased osteopontin levels, which target hepatocytes to promote cholesterol formation and contribute to a chronic inflammatory state.^69^ In the elderly, OPG levels may become dysregulated, diminishing their capacity to inhibit RANKL, thereby increasing bone resorption. This process contributes to osteoporosis and destabilizes the release of other bone-derived hormones, such as osteocalcin, affecting glucose metabolism.^70^ The endocrine functions of aging bone change significantly, resulting in a detrimental effect on whole-body glucose metabolism. Decreased osteocalcin and increased osteopontin and FGF23 levels contribute to insulin resistance, chronic inflammation, and impaired glucose tolerance. Dysregulated OPG accelerates bone resorption rates and affects metabolic health. These changes contribute to the heightened risk of metabolic disorders, such as type 2 diabetes mellitus (T2DM), commonly observed in the elderly.

Herein, we described the pathophysiological alterations of degenerative bone in the aspects of cell, matrix, microenvironment, macrostructure, mechanical properties and physiological function. These alterations should be considered during the design of smart biomaterials and can serve as targets and internal stimuli for smart materials to perform their functions.

### Pathological disorders of skeletal aging

Osteoporosis is the most common pathological aging. Increased bone fragility and fractures are the results of osteoporosis, a degenerative systemic disorder of the skeleton marked by reduced bone mass and imbalanced skeletal microarchitecture.^71,72^ Bone fragility is influenced by bone mass, shape, architecture, and bone quality. The main pathogenetic factors as aforementioned, including cellular senescence, oxidative stress, estrogen deficiency, and genetic elements of skeletal aging contribute to the development of osteoporosis.^71^ Surgery is the main therapeutic option for fracture reduction and immobilization. However, optimal reduction and rigid fixation are difficult to conduct for the comminuted and compromised tissue at osteoporotic fracture sites.^73^ Therefore, the secondary healing process, including inflammation, hematoma, callus formation, and remodeling commences, which is denominated in the fracture healing of skeletal aging. Inflammatory cells, such as neutrophils, B/T cells or macrophages/monocytes, are recruited and accumulated at the hematoma sites. Upon activation, they produce inflammatory cytokines into the local and systemic circulation, including interleukin-1 interleukin-6, and tumor necrosis factor-α,^74^ for pathogen eradication, increased blood flow, and vascular permeability are initiated by these cytokines.^75,76^ Inflammation modulation is vital to trigger the healing pathway and recruit all the necessary elements engaged in the initial repair of the fracture break for indirect bony unions with the absence of rigid fixation.^77^ Thus, regulating various inflammatory cells and cytokines can enhance the healing process of skeletal aging.

OA is considered another age-related disorder, marked by intricate lesions throughout the synovial joint. The pathogenic features include tissue hypertrophy, destabilization of the tendons and ligaments, lack of intact subchondral bone, elevated synovial vascularity, and structural defects in hyaline articular cartilage.^78,79^ Articular cartilage is the structure affected throughout the entire involved OA joint. Research utilizing articular chondrocytes indicates that oxidative stress is increased in aged cells, leading to the development of cell senescence and alterations to mitochondrial activity.^80,81^ Reduced repair response is an additional trait of aging chondrocytes, which is attributable to changes in the receptor expression pattern. The elevated ratio of transforming growth factor-β (TGF-β) receptor activin receptor-like kinase 1/5 in chondrocytes from OA and aged cartilage caused the TGF-β pathway to be down-regulated and the production of catabolic MMP to take over from matrix synthesis activity.^82,83^ The avascular and aneural characteristics of articular cartilage and the intricate pathogenesis of the disorder pose a great problem to its therapeutic strategies.^84^ The surgical regenerative option and conventional palliative pharmacotherapy of these two diseases meet their limitations. Various fragilities and fractures require addressing with appropriate regenerative repair strategies. The related pathological characteristics can act as the markers or evaluation indicators to indicate the treatment.

Skeletal disease is the main cause of significant morbidity and functional decline in old age. Skeletal aging refers to a state of progressive decline and deterioration of bone mass and quality containing natural degeneration and pathological conditions (osteoporosis and osteoarthritis), bearing specific physicochemical changes. These physicochemical characteristics can be used as biomarker profiles to guide the targeting of therapeutic interventions. Thus, it is sufficient and reasonable to adopt smart materials that can respond to these physicochemical features to repair skeletal aging.

## Systemic smart delivery system to improve the overall state of skeletal aging

**Fig. 3:**
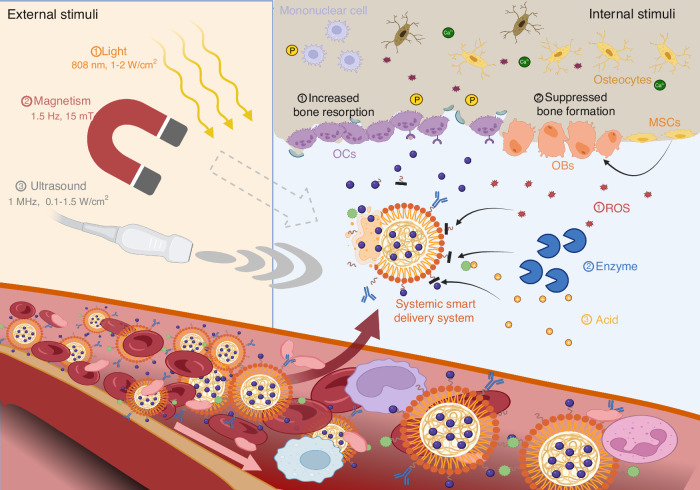
The internal and/or external stimuli activate the systemic smart delivery system to release drugs contributing to bone repair. The systemic smart delivery system patrols the circulation and moves into the degenerated bone microenvironment. The internal stimuli derived from pathophysiological bone tissue include accumulated ROS, specific cleavage enzymes, and an acidic environment, which activate the systemic smart delivery systems. Together with them or alone, the external stimuli, including light, ultrasound, mechanical force, and magnetism, elicit the conformation change and phase transition of the delivery system to release drugs for bone repair and regeneration. OBs: osteoblasts, MSCs: mesenchymal stem cells, OCs: osteoclasts. Created by BioRender software (biorender.com)

The term “smart” represents the specific and predictable responsive manner of systemic delivery systems under endogenous/exogenous stimuli.^85,86^ With the advances of pharmaceutics and materials science, multitudinous bioactive factor cargos, such as liposomes and polymers, have been developed for the improvement and repair of skeletal aging.^87,88^ Time-release, site-specific and dose-controlled drug delivery were developed due to the emergence of surface functionalization methodologies.^89^ The toxicity characterization, pharmacokinetics and pharmacodynamics can be modified by the smart delivery system.^90^ Based on the specific pathophysiological features of the aging skeleton, the smart systems can exploit synergistic effects from various loaded medications to inhibit osteoclast activity and enhance the proliferation and differentiation of osteoblast lineage cells, promoting bone regeneration (Fig. 3). The key parameters including stimulus, activation mechanisms, advanced properties and translation obstacles are summarized in Table 1.

**Table 1:** Comparative analysis of stimulus-specific smart biomaterials for bone aging repair and regeneration

Types of smart biomaterials	Stimulus	Activation mechanism	Advanced properties	Translation obstacles	Ref.
Internal stimuli-responsive smart drug delivery system	pH	Protonation of cleavable groups and gas-destroying particles	Tunable drug release kinetics, pH-triggered charge for cellular uptake, controllable lysosome degradation	Cytotoxicity from hydrazone bonds, autophagy and death from proton-induced endosomal/lysosomal escape, instability	^320^
	ROS	Break of the redox-sensitive bonds (disulfide bonds)	Precise drug release in diverse cell compartments, eliminate related oxidizing species upon activation	Highly tailored formulations for various disorders, counteract self-induced ROS, various factors affecting materials sensitivity	^321^
	Enzyme	Enzyme-cleavable crosslinker	Highly sensitive biometric response, pathological guided drug release	Unclear enzyme content changes, boundary design for normal and pathological situations	^322^
	Ion	Ion exchange and chelation	Stable structure with low cytotoxicity, high surface area	Limited basic research	^323^
	Immune	Interact with the immune signals	Rapid response to the specific immune environment	Further confirmation on the lowest effective concentration	^126^
External stimuli-responsive smart drug delivery system	Heat	Phase transition upon LCST	Precise control of location and intensity, and sequential stimuli	Lack of safe and sensitive materials to detect slight temperature changes near 37 °C	^324^
	Light	Photoisomerization, photothermal, photocleavage and photopolymerization	Noninvasive, tailored exposure time and tissue location	Limited site-specific parameters on delivery depth and focus, UV may induce carcinogenesis, NIR can cause thermal injury	^325^
	Ultrasound	Cavitation phenomena, radiation forces	Safe, cost-effective, energy-focused, deep penetration, and easy to operate	Highly attenuated by bone, difficult to balance the stability and sensitivity, DNA damage	^326^
	Force	Covalent bond cleavage, conformational changes	Self osteo-inductive effect	Unclear optimal mechanical parameter, lack of noninvasive application	^327^
	Magnetism	Magnetic hyperthermia, actuation	Magnetic guidance directs to specific targets, minimal physical interaction with the body, penetrates deep	Constrained magnetic field geometry, complex external magnetic field setup and local overheating from magnetocaloric effect	^328^
Multi-stimuli responsive smart drug delivery system	Dual internal stimuli	Structural transformation	Display superb antioxidative activity and anti-inflammatory effects	Limited quantitative data on the microenvironmental signal	^156^
	Internal and external stimuli	Cleavable bond and electrostatic interactions	Precisely-controlled drug release, activatable imaging-guided therapy	Lack of long-term effects, adverse effectsfrom external stimuli	^158^
Internal stimuli- responsive smart scaffold	Heat	State transition upon LCST	Injectable hydrogel, shape adjusted	Lack of precise control over temperature changes, potential toxicity	^329^
	Ion	Chelation equilibrium shifts	Rapid response, modified bone regenerative environment	Insufficient action duration and intensity	^330^
	pH	Neutralization	Rapid environmental response, modifies local favor acidity, programmable pH response range	Insufficient duration of action, prolonged acidity may hinder regeneration, unintended activation due to physiological pH changes	^331^
	ROS	Chemical bond cleavage and structural degradation	Customizable for disease-specific activation, precise delivery in response to pathological conditions	Limited action range and short lifespan, damage normal cells, oxidative stress	^220^
	Enzyme	Cleavable peptide sequences	High substrate specificity, precise control of release, compatibility	Complex and specific process, balance issue between stability and biodegradability	^224^
	Immune	Immune-related markers	Significant tissue regeneration effect	Unrestricted macrophage activation	^332^
	Glucose	Reversible covalent bond formation	Effectively use excess blood glucose to restore impaired bone remodeling	Whether blood sugar promotes osteogenesis remains unclear	^333^
External stimuli- responsive smart scaffold	Light	Photochemical cleavage and photothermal conversion	Noninvasive with high controllability; versatile applications like drug release, and photothermal effects	Low tissue penetration, damage surrounding normal tissues, potential toxicity from photoactivated materials, photobleaching	^334^
	Ultrasound	Inertial cavitation and sonoporation	Noninvasive and remarkable tissue penetration depth, no drug resistance, precise spatiotemporal control	Low in vivo stability and potential toxicity of sonosensitizers, thermal tissue damage	^335^
	Magnetism	Mechanical actuation and hyperthermia	High tissue penetration, noninvasive with precise control	Non-uniform magnetic heat distribution, high local heat to the surrounding tissue	^336^
	Piezo-electricity	Electromechanical coupling and ion channel activation	Enhanced conductive properties, remarkable self-regenerative effect, mechano-electrical coupling mimics natural mechanical stimulus response	Synthesis issues on densification, alkali volatilization and high temperature, uncertain long-term biosafety and cytotoxicity, limited research on hard tissue repair and regeneration	^337^
	Electricity	Electroporation and electroosmosis	Remarkable regeneration effect without extraneous bioactive molecule	Uncertain cytotoxicity, biocompatibility, and biodegradability, low precision in control	^269^
Multi-function responsive smart scaffold	Dual internal stimuli	Cleavable linker and structure degradation	On-demand release fashion, modulate regenerative microenvironment	Uncontrollable reaction rate	^270^
	Dual external stimuli	Cleavable linker and structure degradation	Synergistic effect for bone regeneration	Inconvenient for activation	^271^
	Internal + external stimuli	Cleavable linker and structure degradation	Allow for sustained or burst and spatial-temporal release	Cost and complex process	^272^

### Systemic smart delivery system response to internal stimuli

#### pH responsive delivery vehicles

Under physiological or pathological conditions, the pH values in various body compartments exhibit distinct ranges. In infected, inflammatory, and diseased tissues, angiogenesis and metabolism are dysfunctional and unbalanced, which leads to the abrupt lack of nutrients and oxygen and a trend to glycolytic metabolism, causing a pH decline.^91^ The diversity of pH in distinct compartments can trigger the activation of the delivery system, which depends on the adaptation of crosslinking processes. In the targeted cell/tissue with a recognizable pH level, an optimal release profile can be obtained through protonation or deprotonation of basic/acidic groups within the material and beneficial factors. Differences in pH in intracellular compartments, tissues, and organs are considered a motivator to induce the release of the beneficial factors at a target site from delivery systems.

**Fig. 4:**
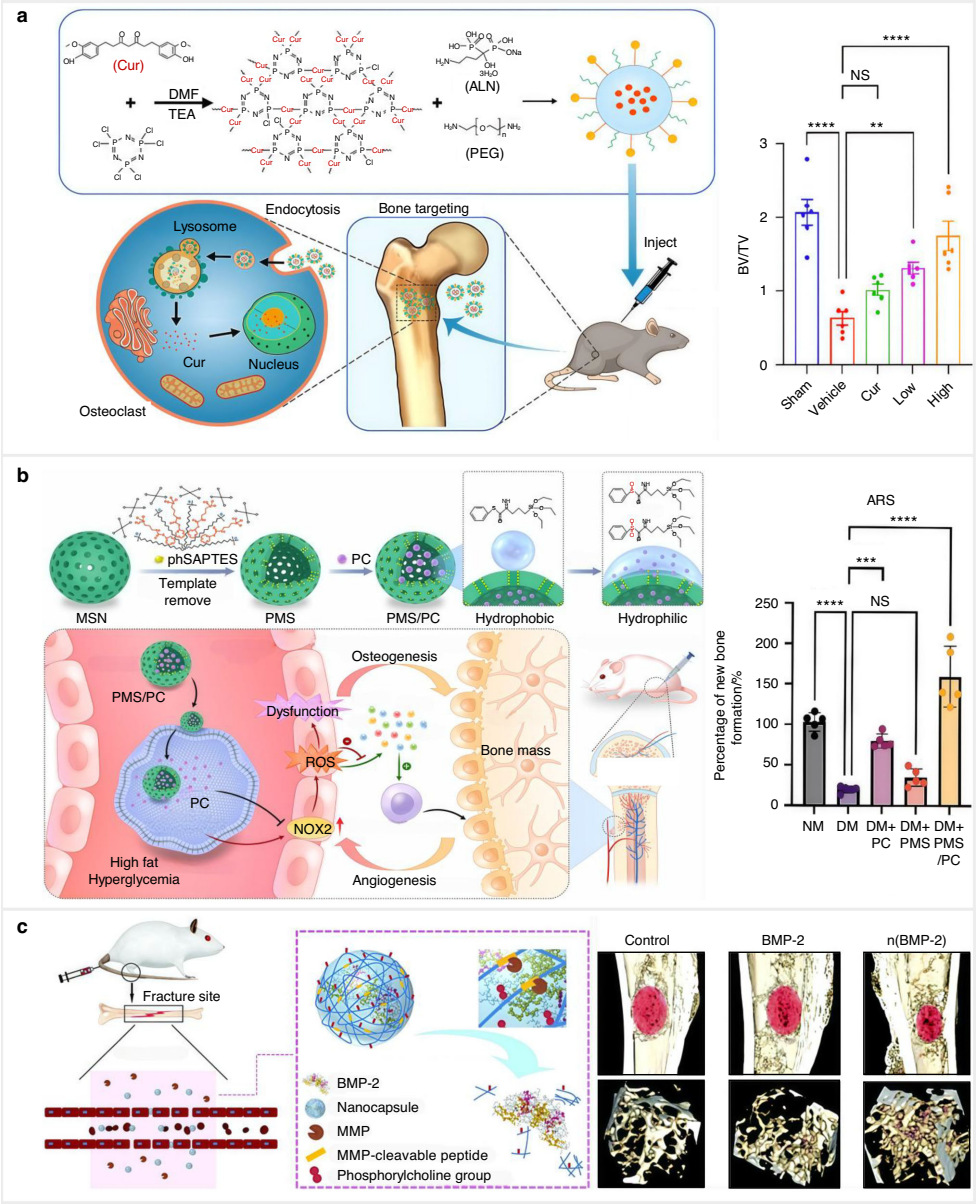
Representative illustration of classic examples of smart drug delivery systems in response to internal stimuli. **a** Schematic illustration of the fabrication process and pH-responsive mechanisms of HCPA nanoparticles for OP treatment. The high dose of HCPA NPs group demonstrated better therapeutic effects on bone loss in ovariectomized mice. Reproduced with permission.^95^ Copyright 2023, Elsevier. **b** Schematic representation of the ROS-responsive PMS/PC delivery system, highlighting its role in promoting the effective coupling of angiogenesis and osteogenesis for new bone formation. Quantitative results of ARS and Masson staining showed ideal effects of PMS/PC on bone formation in vivo. Reproduced with permission.^106^ Copyright 2022, Elsevier. **c** Schematic depiction of the systemic delivery and osteogenic function of MMP-responsive MBP-2 nanocapsules. Reproduced with permission.^114^ Copyright 2019, Royal Society of Chemistry. BV/TV: bone volume relative to total tissue volume, Tb.N: trabecular number

The pH of aging bone resorption lacuna drops to 3–4 due to inflammation and excessive bone resorption.^92,93^ The reduced pH in the aging skeleton can be considered as an internal stimulus to deliver and release therapeutic agents. There are three main fabrication processes for pH-stimulus biomaterials to repair skeletal aging.^94^ 1) Nanocarriers encapsulating bioactive factors: the pH-sensitive release characteristics of the nanocarriers are contributed to the protonation of ionizable groups or the function of pH-responsive cleavable groups. An original skeletal-targeting drug self-framed delivery system was constructed by hexachlorocyclotriphosphonitrile (HCCP), connecting with alendronate (ALN), curcumin, and amino group-terminated polyethylene glycol (HCCP-Cur-PEG-ALN, HCPA NPs). Under an acidic environment, Cur can be released from HCPA NPs and the products exhibit excellent bone-targeting effect and effective inhibition of the proliferation and function of osteoclasts. In an OVX mouse model, HCPA NPs outperformed Cur in terms of anti-bone loss, independent of the dosage. In contrast, the high-dose group exhibited superior therapeutic results compared to the low-dose group, indicating that HCPA NPs had dose-dependent bone regenerative effects (Fig. 4a).^95^ 2) Drug delivery via pH-responsive linkers (such as hydrazine, acetal, and polyketal): for instance, a hyaluronic acid-dependent carrier cargo connected osteoanabolic adenosine with the pH-responsive ketal group. In an OVX mouse model, systemic administration of the adenosine-based delivery improved bone quality and alleviated bone loss.^96^ 3) Gas-destroying particles, containing small substances that can react with acids to produce gases and release therapeutic medicine: for example, ammonium bicarbonate (NH_4_HCO_3_) combined with Poly (lactic-co-glycolic acid) (PLGA) nanocarrier interacts with hydronium ions (H_3_O^+^) to generate gas (CO_2_ and NH_3_). The released gases can break down the shell of nanoparticles, resulting in drug delivery.^97^

#### ROS responsive delivery vehicles

As skeletal aging progressed, malfunctioning mitochondria and repeated stressful loading on damaged bone led to an increase in ROS generation and/or a reduction in antioxidants.^98^ High levels of ROS impair osteoblast lifespan and ECM deposition, resulting in decreased bone mineral density and encouraging the onset of osteoporosis.^99^ Therefore, detecting the ROS level and eliminating overexpressed ROS could alleviate age-related bone loss.

The ROS are highly reactive oxygen-containing chemical species, including singlet oxygen (^1^O_2_), superoxide (O_2_^-^), hydroxyl radicals (OH), and peroxides (H_2_O_2_).^100^ High levels of endogenous ROS are employed as an indicator in stimulus-sensitive skeletal repair and regeneration. Several recent studies have concentrated on this approach and used it to develop synthetic, versatile biomaterials. There are two main mechanisms of ROS-sensitive drug delivery strategies: structural degradation and framework transformation.^101^ Framework transformation represents the ROS-sensitive linker conjugates the drugs or associates with hydrophobic and hydrophilic elements for the physical property alterations.^102^ The soluble properties, including tellurium- and selenium-containing polymers, thioether-containing polymers, and poly(propylene sulfide), could be influenced by ROS levels, favoring the delivery of therapeutic agents.^103^ For example, water-soluble sulfones and hydrophilic sulfoxides can be converted from hydrophobic sulfides under the oxidative environment, which can be used to deliver drugs.^104^ Except for the ROS-based solubility transformation, the oxidants have the potential to break down the chemical connection of phenylboronic acid, poly(proline), poly(thioketal) (TK), and ester-containing polymers, which promote the structural degradation.^105^ ROS-sensitive proanthocyanidin (PC) loaded phenyl sulfide mesoporous silica nanoparticles (PMS) were created to abolish oxidative stress and ROS overproduction in diabetic bone. In vitro and in vivo models showed that the PMS/PC system attained ROS balance by dynamic regulation and promoted osteoblastic differentiation and improved ossification by inhibiting nicotinamide adenine dinucleotide phosphate oxidase 2 (NOX2) (Fig. 4b).^106^

#### Enzyme responsive delivery vehicles

Enzymes are selective and tailored agents that regulate various biological activities, including the production of cytokines and the generation of cell adhesions.^107,108^ Various enzymes are imbalanced in the bone pathological microenvironment during particular pathological or tissue remodeling/repair processes, which can be recognized as a biological stimulus for responsive deliverers to perform diagnostics, medication targeting and release, tissue repair, and regeneration.^109–111^

The classic enzyme employed in an enzyme-responsive delivery system are glycosidases, proteases, lipases, oxidoreductases and phospholipases.^112,113^ The upregulation of MMPs in the microenvironment is an indicator of excessive osteoblastic activities, such as bone aging. Qi et al.^114^ created bone morphogenetic protein-2 (BMP-2) nanocapsules by in situ polymerizing an MMP-cleavable peptide crosslinker and 2-(methacryloyloxy) ethyl phosphorylcholine monomer on the surface of BMP-2, which addresses the problems with the local distribution of growth factors to complicated remodeling microenvironments. MMPs degrade the crosslinker, which leads to polymer shell destruction and release of BMP-2 for skeletal repair and regeneration (Fig. 4c). Apart from enzyme-cleavable junctions, another method for creating enzyme-responsive drug delivery vehicles is enzyme-interactive backbone encapsulation. Tartrate-resistant acid phosphatase (TRAP) is deposited by osteoclasts and builds up in the degenerated bone microenvironment during bone remodeling. To create TRAP binding peptide (TBP)-modified nanoparticles (TBP-NPs), Xiao et al. recently added the TBP to Wnt agonist-loaded poly(styrene-alt-maleic anhydride)-b-poly(styrene) (PSMA-b-PS) nanoparticles.^115^ Based on in vitro results, the TRAP affinity of TBP-NPs peaked at a ligand concentration of 200 000 TBP ligands/NP and was associated with ligand density. It has been confirmed that TRAP-TBP binding caused TBP-NP bone accumulation by in vivo tests using a calcium-deficient diet model, which revealed a significant connection between TRAP deposition and TBP-NP accumulation.

#### Ionic responsive delivery vehicles

The ionic composition of various biological fluids and compartments typically exhibits significant differences.^116^ Specific ions are often released in distinct pathological contexts. Consequently, the unique profiles of pathophysiological electrolytes can serve as identified biomarkers for diverse diseases and can be harnessed to stimulate skeletal repair and regeneration. In certain bone pathological microenvironments, such as osteoporosis and bone defects, there can be an excessive release of calcium ions (Ca^2+^) due to overactivated osteoclast.^117^ The elevated Ca^2+^ levels in bone disease could serve as an initiator. Structural properties of substances such as hyaluronic acid (HA) are significantly influenced by the concentration of Ca^2+^.^118^ The calcium ion-based sheath may firmly enclose the polymer backbone of HA.

For Ca^2+^ responsive drug delivery vehicle, Jiang et al. created the drug-loaded adenine-based metal-organic framework (ZJU-64-NH_2_ MOF) via a solvothermal process. The framework encapsulated the cationic drug procainamide (PA) by electrostatic connection, with 12.97 wt% loading capacity. By illustrating that ion exchange caused the rapid release of PA to rise with increasing Ca^2+^ concentration, the framework demonstrated intriguing bone-target potential. This study creates a novel pathway for Ca^2+^ responsive delivery in degenerated bone treatment.^119^ In another study, high inorganic phosphate ions have been used as a stimulus to trigger drug release for bone therapy. The microenvironment of bone metastasis is characterized by elevated levels of inorganic phosphate ions, resulting from the dissolution of alkaline bone mineral HAp by osteoclasts. A novel nanomedicine comprising phosphate ion-responsive and calcium peroxide-based nanoparticles has been developed. These nanoparticles are surface-functionalized with the bone-targeting agent zoledronic acid and encapsulate the photosensitizer indocyanine green. In the mouse model, these nanoparticles were shown to efficiently accumulate at metastatic bone sites, react with free phosphate ions, and form HAp nanoaggregates. This process promotes the remineralization of the collagenous bone matrix and induces tumor cell apoptosis.^120^ This study illuminates the potential for developing a novel, safe, and efficient therapeutic strategy for treating breast cancer bone metastasis.

#### Immune responsive delivery vehicles

The dynamic balance of bone metabolism is maintained in the bone microenvironment by the communication between immune cells and bone cells via a range of cytokine networks and signal pathways.^121^ Changes in the immunological microenvironment occur in aged bone with the progression of certain bone disorders.^122^ The recruitment and development of osteoblasts may be affected by immune cells such as macrophages and inflammatory cytokines like IL-4 and TGF-β.^123^ Prolonged immune cell and proinflammatory cytokine infiltration into the joints during the pathophysiological state promotes the development of OA.^124^ And OA may eventually arise from a disturbed equilibrium between inflammation and cartilage degradation.^125^

Nanocarriers can be engineered to interact with the particular inflammatory atmosphere and change cell activity to elicit the appropriate immune responses. Xiao et al. delivered the glycogen synthase kinase-3β (GSK3β) inhibitor AR28 into the fracture-associated macrophages using the TBP-NP method. The increased M2 macrophage polarization and improved osteogenesis were demonstrated by an in vitro preosteoblast-macrophage co-culture experiment. As compared to controls, longitudinal examination of TBP-NPAR28-mediated fracture healing showed an elevated M2/M1 ratio and different locations of M2 macrophages.^126^ In another study, a nitric oxide (NO) nanosensor was created to forecast the onset of OA by excessive production of the inflammatory component NO. The nanosensor is composed of NO-sensing molecules and biodegradable PLGA nanoparticles. The NO level in joint fluid of OA rat was quantified and monitored by the nanosensor, which provided cues to evaluate the progression of skeletal aging in real time and without intrusive procedures.^127^

### Systemic smart delivery system response to external stimuli

#### Thermoresponsive delivery vehicles

The external temperature stimulus is achieved by high-intensity focused ultrasound, microwave hyperthermia and radiofrequency thermal ablatio, which creates the temperature disparity between the normal tissue and targeted site. The physical and chemical characteristics of thermoresponsive nanocarriers at the target location can change significantly upon reaching the low critical solution temperature (LCST), which can release the loaded medicines. While the materials should stay stable at 37 °C in normal tissues and sensitive to and responsive to small temperature changes to deliver drugs in locally heated tissue. Key temperature-sensitive components of the biomaterials contains as poly (Ninylisobutyramide) (PAMAM), poly (2-oxazoline) (POxs), poly (N-isopropyl acrylamide) (PNIPAM) and poly [2-(2-methoxyethoxy) ethylmethacrylate] [PMEOMA].^128^

The temperature difference between degenerated bone and normal tissue triggered externally has been explored to develop thermoresponsive smart delivery systems for skeletal aging applications. Poh et al. loaded the anti-inflammatory peptide KAFAKLAARLYRKALARQLGVAA (KAFAK) onto PEGylated poly(N-isopropylacrylamide-2-acrylamido-2-methyl-1-propanesulfonate) particles cross-linked with degradable disulfide (N, N′-bis(acryloyl) cystamine) (NGPEGSS). The addition of PEG and disulfide cross-links preserved the nanoparticles’ thermoresponsiveness, with an LCST of around 35–43 °C. To guarantee that the high load efficiency of KAFAK when the particles were swelled below the LCST, NIPAm was utilized as the polymer backbone. The peptide stayed encapsulated and shielded from proteases when above the LCST and in physiological environments. Drug release profiles revealed that the treatments spatiotemporally controlled release of KAFAK is dependent on the local temperature difference, which was facilitated by the internal stimuli (pH and redox).^129^ In contrast, the cold stimulation can also trigger drug release and promote anti-inflammatory effects. To administer kartogenin and diclofenac, Kang et al. developed thermoresponsive polymeric nanospheres based on chitosan oligosaccharide linked to Pluronic F127 with a grafted carboxyl group. Upon the temperature changes, it can release two drugs independently and simultaneously. The rapidly released DCF was put into the inner core of the nanosphere, while constantly released KGN was covalently cross-linked to the outer portion. The in vivo study revealed decreased level of cyclooxygenase-2 in the serum and synovial membrane, exacerbating by cold treatment.^130^

#### Photoresponsive delivery vehicles

Light is considered an outstanding stimulus, which can be targeted to a given region within 1 µm.^131^ The less invasive approach can be exerted and administered with a highly precise amount, providing temporal and spatial regulation. Various wavelengths of light from near-infrared (NIR) to ultraviolet (UV) have been utilized as triggers. Although UV light is used in stimuli-responsive materials, it can be detrimental and cannot enter tissues deeply. NIR is less harmful to biological tissues, and it is more appropriate for biomedical applications.^132^ Enhanced cell metabolism and elevated ATP production from the mitochondrial membrane can be generated by the photophysical effects of imposed NIR radiation through intracellular behavior effects and respiratory chain improvement.^133^ These behaviors can promote bone repair and angiogenesis.

**Fig. 5:**
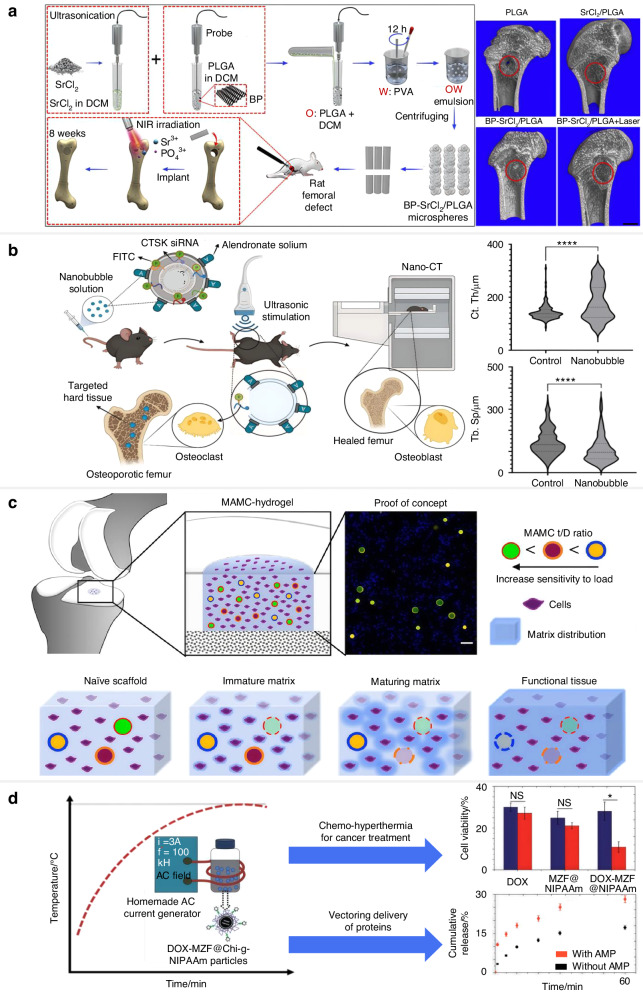
Representative illustration of classic examples of smart drug delivery systems in response to external stimulus. **a** Schematic illustration of the preparation process and NIR-sensitive mechanisms of BP-SrCl_2_/PLGA microspheres for bone regeneration. Reproduced with permission.^135^ Copyright 2018, Elsevier. **b** Schematic illustration of an ultrasound-responsive nanobubble for the targeted delivery of CTSK siRNA to mitigate bone resorption in osteoporosis. Reproduced with permission.^141^ Copyright 2025, Elsevier. **c** Schematic illustration of the biological function of mechanoresponsive microcapsules. Reproduced with permission.^147^ Copyright 2019, Wiley-VCH. **d** Illustration of the MZF@Chi-g-NIPAAm to release drug particles by a magnetic simulant in the fields of cancer therapy and tissue regenerative medicine. Reproduced with permission.^155^ Copyright 2020, Royal Society of Chemistry

Light-responsive systems can be categorized into four groups based on the kinds of photochemical reaction involved: 1) Photoisomerization refers to structural changes induced by light; 2) Photothermal system dissipates the absorbed photon energy via vibrational motion; 3) Photocleavage breaks covalent bonds via the incident light; 4) Photopolymerization reaction enables in situ light-induced cross-linking of composites.^134^ In these regimes, photothermal exerts a dominant role in smart drug delivery for aging skeletal repair. Wang et al. loaded strontium chloride (SrCl_2_) and black phosphorus (BPs) into PLGA to construct BP nanosheets poly (lactic-co-glycolic acid) (BP-SrCl_2_/PLGA) microspheres. The delivery of Sr^2+^ activated by NIR radiation caused localized temperature increases to make leakage in the PLGA shell and promote bone regeneration (Fig. 5a).^135^ A novel natural phenolic acid-based nanohybrid was fabricated to exhibit tunable photothermal effects under mild NIR irradiation for effective suppression of osteosarcoma and promoting bone healing. These nanohybrids can enhance the expression of heat shock proteins and significantly promote osteogenic differentiation under controllable mild NIR irradiation. Simultaneously, they ingeniously integrate the thermal effect to robustly induce apoptosis and inhibit tumor growth.^136^

#### Ultrasound-responsive delivery vehicles

Unlike visible light, magnetic, and electric fields, sonic fields may effectively travel through complicated and opaque medium and pinpoint specific locations in time and space.^137^ Due to the high penetrative tissue over 10 cm, non-invasiveness, and high controllability, ultrasound is regarded as an excellent external mechanical trigger in smart drug delivery systems for skeletal aging repair.^138^ Ultrasound can promote bone regeneration and upregulate the mRNA level of vascular endothelial growth factor A (VEGF-A) to accelerate the synthesis of bone maturation and expedite mineralization.^139^

Scattering, microstreaming, cavitation and acoustic radiation force are the fundamental technological features that underpin ultrasound functions, which are used to precisely regulate biological components that respond to ultrasound.^140^ The ultrasound-responsive capsule embeds the therapeutic drugs temporarily and then patrols in the circulation and accumulates on the surface of defective bone following the ultrasound wave. Cavitation phenomena or radiation forces play mechanical and/or thermal functions in causing the release of drugs. Pedram et al. fabricated an ultrasound-responsive nanobubble (NB) platform loaded with alendronate (NB-CTSK siRNA-ALN) to transmit gene-silencing CTSK siRNA into the aging bone. Biodistribution studies showed the accumulation of NB-CTSK siRNA-AL in the bone and liver. In vivo results showed that the OVX mice treated with NB-CTSK siRNA-AL had increased distal cortical bone thickness (174.4 μm ± 5.28 μm vs. 144.3 μm ± 10.66 μm) and bone volume fraction (16.5% ± 3.96% vs. 6.55% ± 0.13%). Reduced collagen degradation and downregulated CTSK expression were observed in the staining procedures after 4 weekly sessions of treatment (Fig. 5b).^141^ In another study, Ma et al. used a straightforward anti-solvent technique to produce piezoelectric nylon-11 nanoparticles (nylon-11 NPs). Under the guidance of ultrasound, the nylon-11 NPs enhanced the osteogenic differentiation of dental pulp stem cells (DPSCs), markedly increased the expression of genes linked to osteogenesis, and encouraged the development of calcified nodules. This demonstrated that a nanomaterial based on ultrasound stimulation may effectively control stem cell differentiation.^142^

#### Mechanical force-sensitive delivery vehicles

The mechanical force is vital for bone remodeling and can change the fate of progenitor cells, such as migration, proliferation, and differentiation.^143^ During bone repair, appropriate mechanical stress can activate the osteogenic genes.^144^ Varieties of mechanical cues, such as shear stress and ECM rigidity, can be recognized by the transcription factors YAP and TAZ. They promote the proliferation and differentiation of osteoblasts by transducing the mechanical signals into specific transcriptional regulatory pathways.^145^ Under skeletal aging, the programs lead to the early osteogenic differentiation of BMSCs to control bone regeneration and improve bone formation, improving bone regeneration in pathological conditions (e.g., osteoporosis).

Mechanical stress, including tensile, shear, and compressive forces, can be employed as a stimulus or trigger reaction for the smart drug release system. Shen et al. integrated N-heterocyclic carbene-carbodiimide (NHC-CDI) adducts into a novel flex-activated mechanophore to release NHC when it meets a suitable mechanical load. The aryl carbodiimides within NHC-CDIs contributed to the flex-sensitive mechanism. The C-C bond between the CDI and NHC was broken down under mechanical force to lead to the delivery of the NHC.^146^ In response to the mechanically loaded environment of the joint space, Bhavana et al. created mechanically-activated microcapsules (MAMCs) for providing medications in an on-demand manner. The MAMCs could utilize mechanical inputs from the microenvironment for the delivery of TGF-β3 to promote chondrogenesis of MSCs in response to physiological dynamic mechanical loading. With increased microcapsule breakage and drug release in stiff hydrogels, the designed cartilage model’s results demonstrated a graded mechano-activation across a spectrum of stiffnesses (~25 to 150 kPa). Although this mechanical force-sensitive device was created especially to encourage cartilage repair, it has the potential to be widely used in skeletal aging repair and regeneration, where mechanical loading is crucial. (Fig. 5c).^147^

#### Magnetic-responsive delivery vehicles

Low intraparticle diffusion rate, long-term stability, high loading capacity, high specific surface area, and biocompatibility are the desired characteristics of magnetic-sensitive nanomaterials in tissue regeneration.^148,149^ The magnetic nanoparticles are composed of a magnetic metal core and a functional compatible capping with high modifiability for their size, shape, and coating. The Fe_3_O_4_ NPs are employed as medications for magnetic hyperthermia to enhance osteogenic differentiation.^150,151^ Recent studies suggested that medium magnetic strength (1 mT-1 T) can promote the osteoblast mineralization with alternations in the surface Ca^2+^ transport.^152^

When an alternating magnetic field (AMF) or steady magnetic field is applied, functionalized magnetic nanoparticles can be employed as effective drug-delivery vehicles. AMF is defined as a fluctuation in amplitude with time and the capacity of the AMF to heat magnetic materials makes it perfect for magnetically responsive drug delivery.^153^ Utilizing a magnetic field-responsive approach, several researchers use adaptive multifunctional biomaterials that combine the functions of bone disease treatment and bone tissue regeneration. In a rat distraction osteogenesis model, Jia et al. developed mesoporous silica-coated magnetic (Fe_3_O_4_) nanoparticles (M-MSNs) to assess the possibility of accelerating bone repair. Through the canonical Wnt/β-catenin pathway, the M-MSNs demonstrated impressive osteogenic differentiation of MSCs. Following 4-week local injection of M-MSNs, HE staining images showed different levels of newly produced fibrous-like tissue, cartilaginous tissue and bone within the distraction gaps. Apart from magnetic targeting, the release of therapeutic pharmaceuticals from thermally responsive drug carriers can be regulated using magnetic heating.^154^ Temperature-sensitive chitosan-g-N-isopropylacrylamide (Chi-g-NIPAM) polymer was applied to magnetic Mn-Zn ferrite ((Mn, Zn) Fe_3_O_4_) (MZF) nanoparticles. MZF@Chi-g-NIPAM can release BMP-2 and induce localized hyperthermia in the presence of AMF for bone regeneration (Fig. 5d).^155^ These studies demonstrated therapeutic potential for skeletal aging due to the significant osteogenic effect.

### Systemic smart delivery system response to multi-stimuli

Smart delivery systems can diagnose and treat the overall status of the pathophysiological aging skeleton based on internal or external stimuli. The desired application and versatility in the field of biomedicine can be achieved by the modified physicochemical properties. However, sensitivity to a single stimulus leads to suboptimal navigation and distribution due to the presence of complicated physiological fluids and the aged bone microenvironment. There are more various and intricate stimuli at sites of disorders in vivo. The ability of versatile nanocarriers to pass through successive physiological and pathological obstacles to deliver a variety of therapeutic “payloads” to the intended targets has sparked a lot of interest. These carriers can be co-triggered via various signals in various compartments of organisms (such as circulation, skeletal tissue, cells, and subcellular organelles). The environment of bone degeneration exhibits increased MMP activity, high levels of ROS, and reduced pH as a sub-physiological status. To tackle this limitation, smart delivery systems that respond to multiple stimuli emerge to increase sensitivity and selectivity to pathophysiological conditions and improve drug benefits while facilitating personalized treatment.

**Fig. 6:**
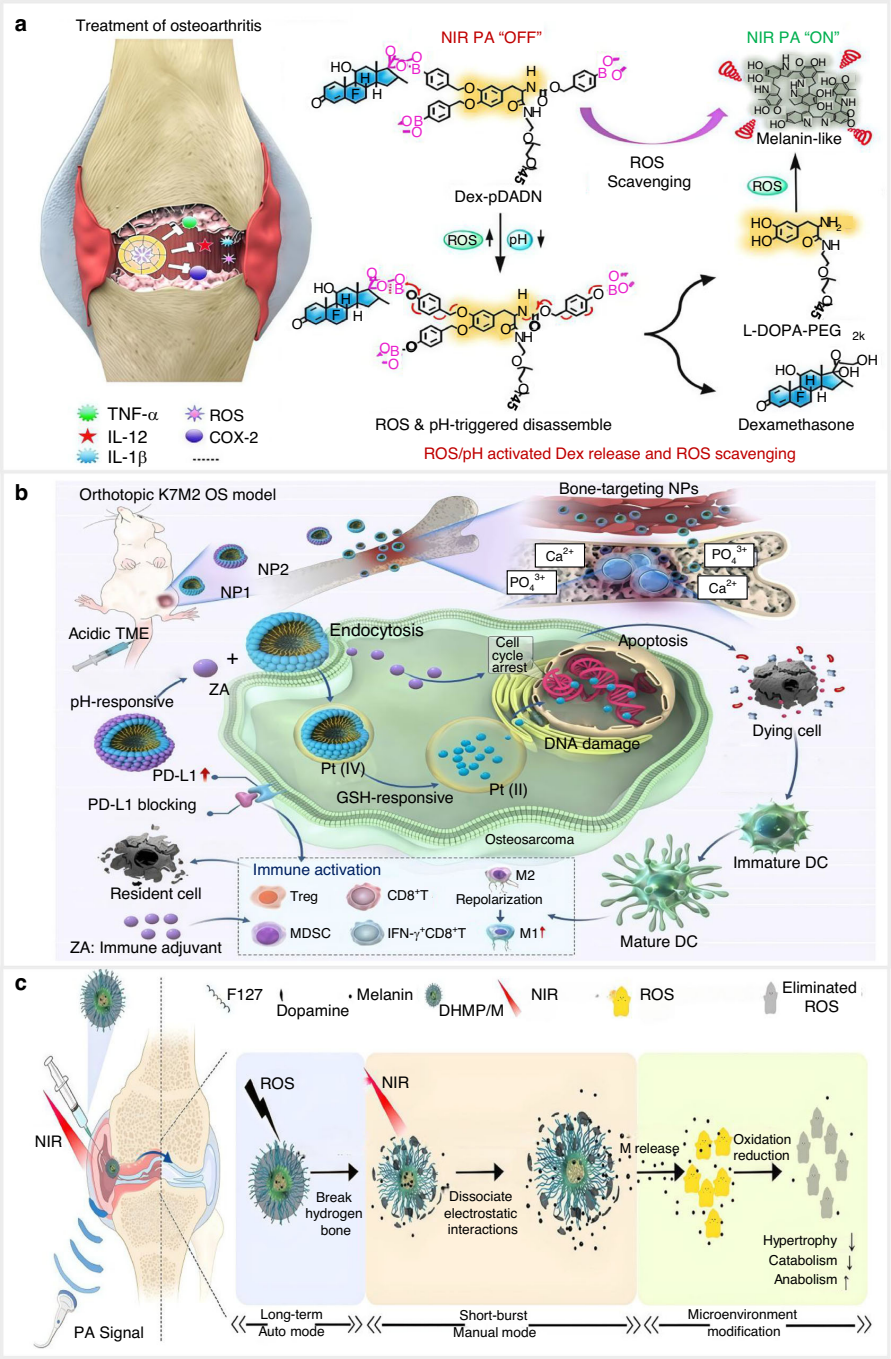
Representative illustration of classic examples of smart drug delivery systems in response to multiple stimuli. **a** Schematic representation of the synthesis of Dex-pPADN for OA treatment, featuring ROS and pH dual-responsiveness for controlled Dex release. Reproduced with permission.^156^ Copyright 2021, Wiley-VCH. **b** Schematic depiction of the fabrication and biological mechanisms of pH and glutathione dual-responsive nanoparticles for enhanced antitumor efficacy. Reproduced with permission.^157^ Copyright 2023, Elsevier. **c** Schematic illustration of the DHMP/M system as a ROS/NIR dual-responsive drug delivery platform for effective knee OA therapy. Reproduced with permission.^158^ Copyright 2021, Elsevier

Smart multi-stimulus-sensitive drug delivery systems can be fabricated by integration of various internal triggers to improve the sensitivity of targeted sites. A pH/ROS dual-sensitive delivery of dexamethasone (DEX) platform was designed by Zhao et al., which consists of self-assembled acid-modified L-DOPA pro-antioxidant (pPAD), phenylboronic and PEGylated nanospheres (pPADN).^156^ Under the ROS, pPADN undergoes conversion to the active form of L-DOPA, transforming into a melanin-like antioxidant substance through a series of oxidative reactions. Concurrently, the loaded DEX was released under the structural transformation of pPADN, potentiated by the acidic pH environment. The in vitro drug release kinetics tests showed that DEX discharged from Dex-pPADN considerably more rapidly at pH 6.5 and/or in the buffer containing H_2_O_2_. After 48 h in an acidic and oxidative environment, ~79% of the encapsulated DEX was released (pH 6.5 + 100 μmol/L H_2_O_2_), compared to 26% of DEX released in a usual physiological environment (pH 7.4). Based on these results, DEX may be released precisely from DEX-pPADN, which would undoubtedly increase medication availability and reduce adverse reactions. The OA rat model verified that the DEX-pPADN treatment groups showed reduced synovial inflammation, inhibited joint destruction, and alleviated cartilage matrix degradation. This finding showed that the framework has intriguing possibilities for the creation of biomaterials to treat aging-related skeletal diseases and enhance glucocorticoid-based inflammation treatment. (Fig. 6a). Targeting acidic immune microenvironment modulation, Li et al. created glutathione- and pH-responsive NPs to deliver zoledronic acid and activate the programmed death ligand 1 (PD-L1) pathway of tumour cells for osteosarcoma treatment (Fig. 6b).^157^

Internal responsiveness, combined with external stimuli-sensitive strategies, is designed to increase selectivity to the controlled time and location. Ruan et al. designed a smart ROS/NIR dual-sensitive release platform under photoacoustic imaging guidance for OA treatment. Under the external NIR stimuli and/or internal ROS, the platform switched the controlled performance of long-term and short-burst release profiles to inhibit cartilage inflammation in the OA rat model (Fig. 6c).^158^ Liu et al. designed a smart pH/NIR dual-sensitive release platform based on TiO_2_ nanotube arrays (TNTs) for osteoporosis treatment. The TNTs were loaded with alendronate and modified with photothermal polydopamine (PDA) and Fe^3+^ (TNTs-PDA-Fe^3+^-NaAL). Specifically, the release of alendronate was preferred at a pH of 4.6 and the photothermal conversion efficiency of the platform was determined to be 32.9%, promoting osteoblast proliferation and differentiation.^159^

To enhance drug-loading capacity and achieve spatiotemporally controlled therapeutic release at target lesions, triple-stimuli-responsive platforms have been engineered for bone regeneration. Cheng et al. developed an acid/NIR/temperature-responsive nanoplatform using yolk-shell periodic mesoporous organosilica nanoparticles (YSPMOs) capped with copper sulfide (CuS). This system endowed the excellent photothermal conversion efficiency. Upon cellular internalization, disulfide bond cleavage in acidic environments combined with NIR irradiation triggered CuS gate removal and DOX release. The synergistic chemo-photothermal therapy significantly enhanced antitumor effect through precise drug spatial control and hyperthermia.^160^

Stimulus-responsive drug delivery systems are emerging as a novel smart biomaterial that can sense single or several internal stimuli (e.g., pH, ROS, enzyme, immune) and external stimuli (e.g., temperature, light, ultrasound, mechanical force, magnetism). They respond to the stimuli by conformational change, solid-liquid phase transition, and other special reactions to release the encapsulated biomolecule with minimal invasion and adverse effects. The drug delivery system can improve drug pharmacokinetics and prolong the patrol time in circulation.^161^ Therefore, the multi-stimuli responsive drug delivery system offers a unique design paradigm for bone treatment by combining conventional therapy with extra controlled release simultaneously.

Despite the attractive advantages and potential of the above systems, various challenges hinder the development of these delivery systems, which is administered intravenously into the circulation system. In addition to the rapid removal of NPs, more than 95% of systemically administered medications tend to pool in organs, such as the liver, spleen, and lungs, with fewer than 5% arriving at the extremely mineralized bone tissue.^162^ When pathological changes occur in localized aging bone, higher NP doses are required for increased drug concentration and accumulation, which results in greater toxicity. Thus, targeted therapeutic strategies are required to treat pathological defects.

## Smart scaffolds for pathological defects

**Fig. 7:**
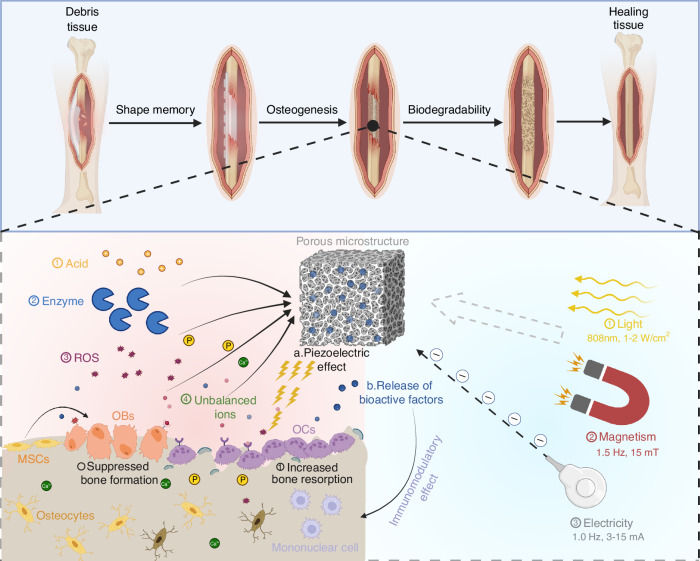
Local smart scaffolds are designed to regenerate aged bone tissue with disorders. The insert smart scaffolds possess the desired porous microstructure and various physicochemical properties such as mechanical strength, shape memory, and biodegradability, which can replace bone defects for bone repair and regeneration. Endogenous disease microenvironments contain various stimulatory factors, including an acidic environment, cleavage enzymes, and accumulated ROS, and increased ion (Ca^2+^) to trigger the inserted smart scaffold. Additionally, external strategies contain light, magnetism, and electric signals that could stimulate the temporary scaffold. The activated scaffold releases bioactive factors and exhibits piezoelectric capability and immunomodulatory effect to promote bone regeneration. OBs: osteoblasts, MSCs: mesenchymal stem cells, OCs: osteoclasts. Created by BioRender software (biorender.com)

The remodeling process and healing performance of bone deteriorate with age. The occurrence of bone defects and fractures increases in aged people with compromised repair ability, which creates a demand for effective therapies promoting bone regeneration. The local smart scaffolds are recognized as tailored structural support that can incorporate bioactive agents to replenish damaged tissue and provide mechanical strength.^163,164^ The released bioactive agents can up-regulate regeneration-related proteins and promote targeted signal cascades to improve cell migration, adhesion, multiplication, and differentiation.^165,166^ The targeted cells can enhance the deposition of ECM and mineralization to regenerate the aging skeleton. Smart scaffolds, which respond to external stimuli or internal stimuli and contribute to the regeneration of skeletal tissues in an operative and meritorious way, are introduced as follows (Fig. 7).

### Physicochemical properties of smart scaffolds

The properties of smart scaffolds are influenced by the selected raw materials and manufacturing techniques. Following raw chemical constituents of the biomaterials, physicochemical parameters, such as stiffness, viscoelasticity, and porosity, affect the performance of bone regeneration. Herein, we discussed the features of engineered smart scaffolds to tailor tissue repair and regeneration processes.

### Raw materials of smart scaffolds

Raw materials selected for tissue engineering resemble the tissue microenvironment, which is versatile and efficient regarding the clinic and cost. Three main types of raw materials are available for constructing smart scaffolds to treat degenerated bone, including metals, bioceramics, and polymers.

The oldest implant materials are metals, which were documented in Egyptian times.^167,168^ Aluminum, lead, gold, and silver were the first metallic materials used for bone repair.^169^ To date, spine, knee, hip, and dental metal implants account for the majority of global implants due to fatigue resistance and high tensile strength.^170^ Titanium and related alloys become the most practiced metallic biometallic materials for orthopedic and dental implants due to the safety, corrosion tolerance, and biocompatibility of TiO_2_ surface.^171^ Degradable metal scaffolds disrupt the stereotype of insoluble metallic materials and are in increasing development. A 3D-printing Zn-0.8 Mg alloy with adaptive biodegradability was fabricated by Xu et al. with favorable mechanical performance and bi-directional regulation of bone metabolism. In the mouse calvarial osteolysis model, the Mg scaffolds activate the PI3k/Akt pathway to facilitate osteogenic differentiation and downregulated the GRB2/ERK cascade to suppress osteoclast differentiation.^172^

Bioceramics and bioactive glasses are used in tissue engineering, especially in dentistry and orthopedics. In the 1970s, Hench introduced bioactive glasses that could bond with both bone and soft tissues in the human body.^173^ Tricalcium phosphate (TCP) and HAp [Ca_10_(PO_4_)_6_(OH)_2_], together with their composites, are the most widely used bioceramics in bone-tissue engineering. These materials can be identified by their low elasticity, brittleness, and high mechanical stiffness.^174^ As a significant naturally existing inorganic component of bone, Hap has remarkable osteoconductivity, bioactivity, biocompatibility, non-toxicity, and anti-inflammatory properties.^175^

Obtained with desirable degradability, current bioactive polymers are employed as tissue engineering scaffolds to simulate some of the local ECM characteristics.^176^ The biodegradable polymers are divided into two categories: synthetic and natural polymers.^169^ Natural polymers, including proteins (e.g., fibrin gels, collagen, soy, and silk) and polysaccharides (e.g., hyaluronic acid, chitosan, starch, and alginate), afford desired cell growth and attachment effects.^177,178^ The structural protein collagen I is present in bone, cartilage, skin, and ligament, which is one of the most employed natural polymers.^179,180^ Polypeptide chains of collagen are mainly composed of glycine, lysine, proline, and hydroxyproline, and their flexibility depends on the proportion of glycine.^174^ Synthetic polymers based on polyesters, including poly ε-caprolactone (PCL), polyglycolic acid (PGA), polylactic acid (PLA), or poly (lactic-*co*-glycolide) (PLGA) copolymers undergo degradation by ester bond hydrolysis and bulk erosion.^181^ Changes to the co-polymer ratio, crystallinity, and molecular weight can alter the degradation rate from weeks to years.^182,183^ In the field of bone engineering, poly (α-hydroxy acids), such as PLGA, PGA and poly (L-lactic acid) (PLLA), are the most utilized synthetic polymers for three-dimensional scaffolds.^169^ Copolymers made of polymers and bioactive ceramics (especially HAp) are receiving a lot of attention and development for bone engineering applications to avoid the adverse effects associated with conventional polymers.^184^

### Physical properties of smart scaffold materials

Mechanical strength is an important factor that must be considered when planning or choosing whether to use a scaffold to treat damaged bones. Targeted mechanical loading not only orchestrates collagen-fiber realignment during ECM remodeling but also directs osteogenic lineage commitment of MSCs, thus accelerating mineralized bone regeneration.^17,185,186^ The mechanical strength of biomaterials aims to harmonize with surrounding tissues at defect sites, providing essential stability until newly formed bone assumes structural roles.^187^ For instance, the elastic modulus, reflecting a scaffold’s load-bearing capacity, should approximate natural bone to ensure mechanical support while preventing stress shielding. Alloy-based scaffolds (e.g., stainless steel, Ti, Fe, Mg, Zn, Ta, and Bi alloys) exhibit elastic moduli ranging from 6.4 to 200 GPa, contingent on composition and fabrication methods.^188,189^ Bioceramic and polymer scaffolds show moduli of 3.0–59.8 GPa and ~2.3 GPa, respectively, aligning with cortical and trabecular aged bone values.^190^ Fatigue resistance, defined as the endurance under cyclic loading, is critical for long-term functionality. Cortical bone achieves 60–70 MPa fatigue strength at 10⁷ cycles, facilitated by its collagen- HAp hierarchy and fluid-mediated nutrient transport. Biometal scaffolds demonstrate superior fatigue strength (150–250 MPa) through bimodal grain structures and work hardening.^191^ While bioceramic and polymer scaffolds exhibit limited fatigue resistance due to stress concentration at pores and molecular chain slippage, leading to limited applications on non-loaded or low-loaded positions. Additionally, enhanced elastic modulus exhibits shape memory properties by preserving the scaffold’s structure and form under repeated and constant physiological pressures. Adequate viscoelasticity facilitates the transfer of mechanical stimuli to regenerated bone and MSCs, which has attracted a considerable amount of research in this area.^192^ For full-thickness osteochondral regeneration, Dan et al. developed a difunctional PEGylated poly (glycerol sebacate) (PEGS)/mesoporous bioactive glass (MBG) bilayer scaffold with adequate viscoelasticity. The viscoelastic PEGS-12 with low crosslinking degree may enhance articular cartilage matrix secretion, preserve chondrocyte phenotype, and encourage chondrogenic differentiation in articular cartilage regeneration.^193^

Porosity is critical for providing sufficient volume for cell migration, proliferation, and vascularization.^194^ Size, geometry, and spatial distribution of pores are the key characteristics that enhance the regenerative efficacy of bone. Macropore scaffolds (150–800 µm) can promote osteogenesis, angiogenesis, and cell accumulation at the inserted site to encourage vascular growth, nutritional transport, bone regeneration, bacterial disposal, and macrophage infiltration. In contrast, smaller pore (<100 µm) promotes the formation of fibrous tissue and non-mineralized osteoid and provide substantial space for protein attachment, cell migration, and adhesion.^195–197^ The scaffold with moderate pore size (500–800 µm) is suitable for bone tissue regeneration since it allows for sufficient cell proliferation.^198^ The geometry of scaffold pores is another factor regulating the rate of bone regeneration. Variations in surface curvature and pore width affect the shape of the tissues and their pace of repair. Compared to long edges, there was a greater cell multiplication around the short margins of rectangular holes. Concave surfaces of scaffolds promote tissue development by allowing sufficient area for cell attachment, while the convex surface restricts the development of tissues.^164,199^ In vitro experiments confirmed that the concave architecture at the pillar intersections promotes interaction between cells and bridging development of BMSCs, improving their adherence and growth on the pillars and permitting bridging growth over the grain boundary structure.^200^

### Shape memory of the smart scaffolds

The shape memory is the ability to change shape in response to an external signal, consisting of shape fixation and restoration.^201^ Most of the shape memory of smart scaffolds is thermo-responsive or hydro-responsive, with shape recovery driven by temperature elevation or hydration. They have received substantial attention as a result of the specific shape-driven capabilities for improved insertion and/or defective tissue filling, with the ability to self-unfolding, self-expanding, or self-sealing.^201^ The small size of the primitive implant allows it to be inserted into the body in a minimally invasive manner with minimal damage to the host tissue. After implantation, the implant returns to a larger shape to fulfill the bone deformity and meet the border of irregular bone deficits.^202,203^ Researchers fabricated BMP2-loaded shape memory porous nanocomposite scaffolds consisting of Hap and poly(ε-caprolactone) nanoparticles to restore defective bone. During the in vitro and in vivo models, the porous insertions showed shape memory recovery from compressed 33 μm pores to the primary porous shape with a diameter of 160 μm. The results of histomorphometry and in vivo µ-CT of rabbit mandibular defects suggested the excellent osteogenesis of the scaffolds.^204^

### Biodegradability of smart scaffold materials

A novel group of biodegradable materials has been developed to meet the advances in regenerative medicine and tissue engineering, which addresses the problem of secondary surgery. Numerous biodegradable synthetic polymers have been utilized in the manufacture of biomaterials over the past few decades, including PLA, PGA, PLGA, and PCL.^170^ Enzymatic and hydrolytic degradation are two common modes. The hydrolytic degradation kinetics are polymer-unique: degradation ratio declines with increasing hydrophobicity, which is a key factor in determining the balance of water diffusion of polymeric biomaterials. The mechanism of chain degradation includes bulk degradation (e.g., accumulation of degraded particles and water within polymer) and surface erosion mechanism (e.g., degradation from the surface), which is determined by degradation kinetics.^205^ The degraded products by bulk degradation may aggregate within the material, but are generated byproducts that affect clinical outcomes. The surface erosion degraded particles can spread into the peripheral tissues or be eliminated by fluid convection.^206^

### Smart scaffolds respond to internal stimuli

#### Temperature responsive scaffolds

The temperatures of certain pathophysiological situations, such as inflammation and fracture, are higher than those of the surrounding healthy tissues. It allows the development of a thermoresponsive smart scaffold for skeletal aging with chronic inflammation. The local temperature of a broken bone may mildly elevate (≈2.0–3.1 °C) due to angiogenesis and inflammatory reaction, which can serve as an internal trigger to activate the scaffold.^207^ The physical characteristics or structure of thermoresponsive scaffolds are designed to undergo reversible changes in response to elevated local temperatures, thereby enabling the controlled release of bioactive factors such as drugs, cytokines, and signaling molecules. This temperature-dependent phase transition is primarily dictated by the hydrophobic/hydrophilic balance and solvent-polymer interaction. Addition of reactants, such as surfactants, plasticizers, co-solvents, and copolymers to the solvent/polymer system can change the thermoresponsive performance of the polymer.^208^

**Fig. 8:**
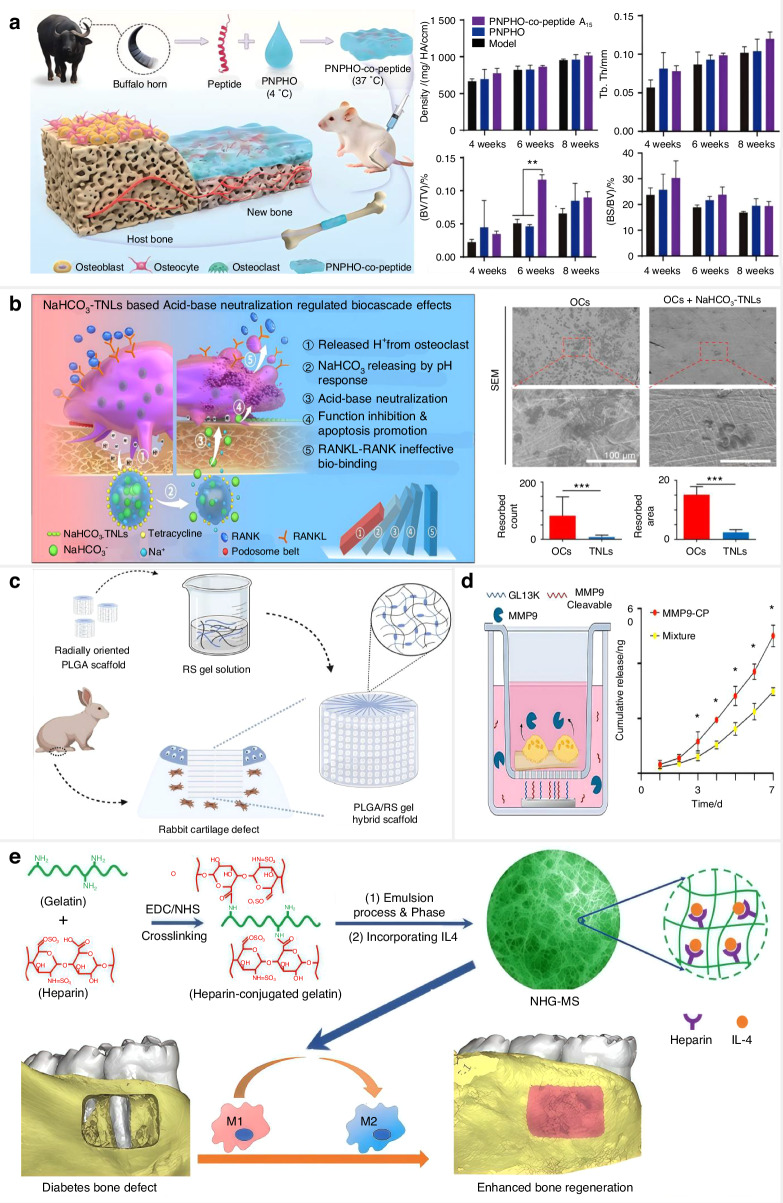
Representative illustration of classic examples of smart scaffolds in response to internal stimuli. **a** Schematic diagram of a thermoresponsive hydrogel incorporating biofunctional buffalo horn peptides to enhance angiogenesis and osteogenesis, combined with the longitudinal repair outcomes of PNPHO-co-peptide A_15_ hydrogel in 8 weeks. Reproduced with permission.^209^ Copyright 2024, Wiley-VCH. **b** Schematic representation of acid-sensitive NaHCO_3_-TNLs targeting osteoclasts for osteoporosis therapy, confirmed by SEM results that NaHCO_3_-TNLs prevented osteoclast acid erosion in the bone resorption assay. Reproduced with permission.^215^ Copyright 2020, American Chemical Society. **c** Schematic depiction of the preparation and structure of a ROS-responsive hydrogel for in situ regeneration of cartilage defects in a rabbit joint model. Reproduced with permission.^219^ Copyright 2021, IOP Publishing. **d** Illustration of the release profile of osteoclast-secreted MMP-9-CP peptides from monolayer-immobilized and mixed peptide coatings. Reproduced with permission.^223^ Copyright 2021, Elsevier. **e** Schematic illustration of the preparation and immunomodulatory effects of IL-4-loaded NHG-MSNs. Reproduced with permission.^239^ Copyright 2019, American Chemical Society. BS/BV: bone surface/bone volume, BV/TV: bone volume/total volume ratio, Tb.Th: trabecular thickness, SEM: scanning electron microscopy

Xue et al. designed biocompatible thermoresponsive poly (NIPAAm-co-(N-acryloxysuccinimide)-co-(polylactide/-hydroxy methacrylate)-co-(oligo (ethylene glycol)) (PNPHO) hydrogel incorporated with horn peptide to promote bone regeneration and angiogenesis for the critical-sized skeletal defects. The temperature and degradation rate of the hydrogel were adjusted by changing the peptide content. Compared to the model group, the average bone trabecular density in the PNPHO-co-A_15_ group increased by 13.66%, 5.36%, and 6.14% after 4, 6, and 8 weeks of administration, respectively. The injected hydrogel rapidly filled the bone defect site, which exhibited a superior angiogenic response and faster bone regeneration with minimal invasion, favoring elderly patients (Fig. 8a).^209^ Furthermore, an injectable thermoresponsive chitosan/silk fibroin (CS) hydrogel composed of BMP-2-functionalized MgFe-layered double hydroxide (LDH) nanosheets and loaded with platelet-derived growth factor-BB (PDGF-BB) can achieve a burst release of PDGF-BB and a sustained release of BMP-2.^210^ In vitro experiments showed that the incorporation of MgFe-LDH in CS hydrogel not only shortens the gelation time and decreases sol-gel transition temperature, but also enhances the mechanical property of the hydrogel. By the end of the 35-day measurement period, the cumulative release of BMP-2 was about 74.51%. In contrast, 80.03% of PDGF-BB encapsulated in the CS hydrogel was released during the initial 7 days. In vivo experiments further prove that the CSP-LB hydrogel can significantly enhance bone regeneration with higher bone volume and mineral density than that of the controls at 8 weeks. These findings indicate the potential of thermosensitive scaffolds as promising candidates for bone regeneration therapy.

#### Ionic responsive scaffolds

In certain specific pathological conditions, such as diabetic wounds, osteoarthritis, and pathological fractures, distinct ionic patterns are established.^116^ These ionic changes can serve as activators for smart materials. Krishna et al. fabricated scaffolds from polyelectrolyte of hyaluronic acid (HA) and chitosan (CH) with bovine serum albumin loaded to investigate the release profile in the presence of Ca^2+^. The level of molecular release was observed in aqueous solutions of Ca^2+^ and Na^+^ and deionized water. The presence of Ca^2+^ increased the release rate.^211^ Additionally, the novel smart biomaterial can induce accurate and efficient repair of defective bones by stimulating the release of growth factors or drugs in Ca^2+^-rich areas, such as bone cracks.

Although ionic concentration-responsive materials remain underexplored, emerging evidence highlights electrolyte imbalances as a pivotal biomarker of skeletal aging. Integrating this innovative approach with cutting-edge material science holds significant promise for developing next-generation precision therapies targeting bone regeneration.

#### pH responsive scaffolds

The normal pH of the body tissue is mildly alkaline, whereas, in certain specific pathologies such as chronic inflammations, the environment of body fluids is weakly acidic.^212–214^ Lin et al. created a smart “nanosacrificial layer” by incorporating NaHCO_3_ into tetracycline-functionalized nanoliposomes. The local acidic condition resulting from aberrant osteoclasts can be targeted by the smart layer, which adheres to the bone surfaces to create an alkaline shield to neutralize the acid. By inhibiting the aberrant osteoclasts, the sequential onset of osteoclast apoptosis facilitates the release of extracellular vesicles, which harbor RANK and RANKL. This mechanism has the potential to treat osteoporosis by reversing bone loss, reshaping the bone microenvironment, and promoting bone regeneration (Fig. 8b).^215^ For skeletal deformities and infections, Deng et al. created a unique “pDA-Ag-pDA” sandwich design that integrates apatite on the following pDA layer and traps Ag NPs on the initial pDA layer. The polyetheretherketone scaffold with acidity-responsive ion release characteristics is made achievable by these special coatings. In the bacterial-infected acidic condition, Ag^+^ is released to kill bacteria, while Ca^2+^ and PO_4_^3−^ are released to promote osteogenesis.^216^ Targeting soft skeletal tissue, Bian et al. developed an injectable, self-healing supramolecular hydrogel based on molecular tautomerism.^217^ The tautomerized cyanuric acid specifically binds to adenine-rich nucleic acids, enabling the enrichment and subsequent pH-dependent release of mRNA. The hydrogel facilitated a sustained release of FGF18 mRNA over 7 days and doubled its in vitro stability compared to free mRNA. The micro-CT analysis of the rat OA model demonstrated a larger bone volume and reduced bone erosion relative to controls. These findings suggest that this pH-responsive strategy represents a promising therapeutic approach for the treatment of skeletal aging.

#### Oxidative responsive scaffolds

Repair of osteoporotic bone defects remains a significant challenge due to insufficient bone regeneration and an abnormal level of ROS, which leads to cell damage, inflammatory responses, and subsequent impediment of normal bone tissue formation. Parallel to the electronegative properties of defective bone, aberrant accumulation of ROS is found in various bone-related disease scenarios, including bone metastases, OA and osteoporosis.^218^ Hydrogen peroxide (H_2_O_2_) and superoxide (O_2_) are the two primary ROS that enhance osteoclastogenesis among these disorders.^105^

The smart scaffold can be designed to be activated in the presence of abnormal ROS accumulation and regulate ROS to rebalance the activation of osteoblasts and osteoclasts.^218^ Wu et al. prepared a novel ROS-responsive hybrid scaffold consisting of a ROS-scavenging hydrogel (RS Gel) and an oriented poly (lactic acid-polyglycol) (PLGA) scaffold. The oxidative response was obtained from the hyperbranched polymers of RS Gel. In vivo experiments showed that after 12 weeks of treatment, the implanted hybrid scaffold suppressed inflammation and promoted hyaline cartilage regeneration (Fig. 8c).^219^ A recent study created a ROS-responsive GelMA hydrogel incorporating METRNL (RRG-MRL) to act as a “bone microenvironment-modulating system” for targeted METRNL delivery. In regions with high ROS levels, the ROS-cleavable NHS-TK-NHS linkers break down, prompting hydrogel degradation and METRNL release. This approach significantly diminished ROS, alleviated inflammation, and boosted anti-apoptotic factors. Released METRNL spurred endothelial cell angiogenesis via the c-Kit/PI3K/Akt pathway and raised SDF-1α secretion to attract BMSCs. Rat cranial defect models treated with RRG-MRL showed lower ROS signals and better neovascularization, accelerating bone regeneration and showing potential for repairing pathological skeletal aging.^220^

#### Enzyme responsive scaffolds

The development of disorders is accompanied by imbalances in different enzymes, such as proteases, lipidases, oxidoreductases, and glycosidases. For instance, in conditions such as OA and osteoporosis, the dysregulation of MMPs is particularly notable. Enzyme-catalyzed biochemical reactions are highly selective for their substrate processes, allowing for specialized and complex processing.^221^ Based on these characteristics, a variety of smart biomaterials have emerged that can localize specific enzymes in cells and tissues in situ to perform a variety of functions, including diagnostics, drug delivery, and tissue repair.^222^

During bone aging, large amounts of MMP-9 are produced during osteoclast-mediated bone remodeling and accumulate around the inserted scaffold for removal. A novel titanium bone implant loaded with co-immobilized surface involving MMP-9 responsive and GL13K antimicrobial peptide was created that responds to high concentrations of MMP and prevents infection. The co-immobilized surface exhibited excellent anti-biofilm performance, enhancing fibroblast and osteoblast proliferation (Fig. 8d).^223^ Alkaline phosphatase (ALP) enzyme is also recognized as the target of stimulus. Liu et al. created an ALP-responsive biodegradable polyphosphoester (PPE) dendrimer, composed of cross-linked poly(propylene fumarate) (PPF) and polycaprolactone (PCL). The dendrimer was able to support stem cell differentiation and bone regeneration. During the bone modelling, the PPE enable polymer cleavage in response to ALP. The scaffold samples in the solution with a low concentration of 100 U/L ALP enzyme showed half weight loss in the region of 160–180 days and demonstrated full degradation in around 260 days. Additionally, the rat calvaria defect model evaluated the bone formation capability of the scaffolds and demonstrated almost complete defect filling and tight bridging between the original bone segments within the defect site.^224^

Despite advances in enzymatic systems, key limitations hinder their bone engineering applications. Patient variability in enzyme expression levels compromises therapeutic consistency. Also, the low specificity of MMPs (e.g., MMP1/8/13 all cleave Gly-Ile/Leu bonds) causes off-target effects. These constraints restrict the clinical translation of enzyme-responsive scaffolds.

#### Immune responsive scaffold

The immune environment of local tissue can be disrupted by the progression of various disorders (e.g., OA, osteoporotic fracture, diabetic foot) and the insertion of different biomaterials, which can induce abnormal development of immune cells and factors. Immune cells that include dendritic cells, macrophages, neutrophils, mast cells, and lymphocytes play a key role in skeletal homeostasis and participate in the regulation of cell aggregation, osteoclastic and osteogenic differentiation, fibrosis, and vascularization.^225^ The immune system is essential in regulating bone remodeling. Following insertion, the biomaterial is recognized by the immune system as “foreign” prompting an immediate reaction. Inflammatory factors are synthesized and combined with activated immune cells to encapsulate or phagocytose the materials for removal.^226–229^

Conventional manufacturing strategies evade the immune response with the development of inert biomaterials.^230^ However, this method caused poor regenerative results due to poor vascular invasion and inadequate nutrient supply.^229,231^ The crucial effect of the inflammatory and immune response of immune cells on tissue regeneration and biomaterial insertion has obtained increasing attention.^232,233^ Activated lymphocytes with increased RANKL enhance the activity of osteoclasts and participate in the process of bone resorption.^122^ The immune system can mediate host-implant communication. Numerous valid immune strategies have been developed to improve the regenerative effect, such as the investigation of the immunomodulatory scaffold, incorporation of inflammatory factors, and application of novel surface layers. They strive to improve the bone immunomodulatory properties of biomaterials to shift the immune environment from bone resorption to regeneration.^234–237^

Over the past decades, smart biomaterials that respond to specific immune environments have shown great promise in the therapeutic field. The osteoinductive microenvironment beneficial for the survival and proliferation of stem cells can be created by smart osteoimmunomodulatory scaffolds.^238^ A novel injectable microsphere was created by Hu et al. that serves as an osteoimmunomodulatory scaffold to regulate macrophages activities in diabetes mellitus (DM) environment. The heparin-modified gelatin nanofibers and the immunomodulatory cytokine IL-4 were both located in the self-assembled microsphere. The microsphere can engage with proinflammatory M1 macrophages in the proinflammatory milieu of DM and give them the M2 phenotype, restoring the normal M1/M2 ratio. This switch can reverse inflammation, promote osteoblastic development, and facilitate osteogenesis.^239^ Thus, this new type of injectable microsphere illustrates an intriguing approach to resolving inflammation and boosting bone repair in a DM environment (Fig. 8e).

#### Glucose responsive scaffold

In certain pathological states, such as diabetes, patients frequently experience delayed tissue healing and disrupted bone metabolism, which can accelerate skeletal aging.^240^ Elevated blood glucose levels induce local microvascular constriction and bone tissue damage, thereby impairing blood supply and oxygen delivery. This cascade of events leads to mitochondrial dysfunction and increased synthesis of ROS and MMPs, all of which exacerbate local inflammatory responses.^241^ Moreover, these alterations can impair the function of key cells involved in bone regeneration, such as BMSCs and macrophages, resulting in compromised bone healing.^242^

Fluctuations in blood glucose levels, often due to inadequate therapy and poor lifestyles, are common in T2DM patients and represent a potential target for smart scaffold activation. A novel hydrogel delivery system (Exos-Smpd3@Ns), comprising Smpd3-overexpressing stem cell-derived exosomes (Exos-Smpd3) and nanosilver ions (Ns), was developed for bone regeneration and repair. The glucose responsiveness of this system is conferred by phenylboronic acid-based polyvinyl alcohol crosslinkers. In vivo and in vitro studies have shown that the Smpd3 delivery system activates BMSC activity and promotes bone regeneration under fluctuating blood glucose conditions.^243^ Additionally, Liu et al. designed a smart glucose-sensitive, sustained-release Resveratrol-loaded hydrogel platform to restore mesenchymal stem cells and scavenge excessive ROS. Resveratrol was immobilized within the hybrid hydrogel through the dynamic borate bond formation made by the grafting of glucose-sensitive phenylboronic acid onto the GelMA chain. By stimulating the PI3K/Akt/GSK-3β signaling cascade, this hydrogel platform significantly reduces inflammation, modifies macrophage polarization, and promotes osteogenesis.^244^ All of these findings point to the possibility that this novel glucose-responsive hydrogel platform offers a theoretical foundation and a possible approach for the treatment of bone defects in individuals with T2DM.

### Smart scaffolds respond to external stimuli

Sustained and stable endogenous stimulation comes from the local pathophysiological environment, whereas some defective sites require more therapeutic intensity and strict time control. Temporal and dosage regulation could be achieved by changing the intensity and activity of the applied exogenous stimuli. Light, magnetism, and electric stimuli are three common external stimuli for smart biomaterial activation. By incorporating photothermal nano-agents, MNPs, or piezoelectric agents into the manufacture of a biomaterial, it is possible to produce scaffolds that respond to common external triggers. The following section describes light-, magnetic-field-, and electrically responsive smart biomaterials, including some examples that have exploited a diversity of external stimuli.

#### Light responsive smart scaffolds

A variety of light-responsive composites-based smart scaffolds are used to modify the surrounding environment, including transition metal dichalcogenides, transition metal oxides (TiO_2_), graphite carbon nitride, gold-based and carbon-based nanomaterials (graphene oxide and carbon nanotubes).^245^ Following light activation, the conformation, hydrophobicity, and/or polarity can be changed to release loaded nano-vehicles and control cell behavior for bone repair.^245,246^

Owing to the short wavelength of UV light, it is employed in light-responsive scaffolds to promote loaded drug release.^247^ DEX is a synthetic glucocorticoid that induces osteogenic differentiation of hMSCs, which is related to a higher risk of osteonecrosis and osteoporosis in terms of long injections. Zhang et al. fabricated a temporal and dosage-controlled DEX-loaded microgel with a photocleavable molecule, O-nitrobenzyl ester, characterized by the membrane-disrupting properties and light-triggered drug liberation. By modulating the light source, the differentiation of hMSCs can be governed, offering a feature amenable to clinical translation.^248^ NIR light shows great biomedical potential due to its precise remote control and noninvasive properties. A 3D-printed biomimetic scaffolding system loaded with two drugs was engineered to facilitate spatiotemporally modulated drug dissemination. This construct recruits host stem cells and drives osteogenic progression through a two-phase process to bone defects. The chemotactic agent simvastatin (SIM) was incorporated into the hydroxyapatite/collagen bioink matrix for swift initial release in the early stages of bone restoration. NIR-responsive polydopamine-coated HAp nanoparticles delivered the osteogenic drug pargyline (PGL). This intelligent scaffold, with its on-demand sequential elution profile, augmented ALP actions, stimulated the upregulation of osteogenesis-related genetic markers, and intensified new bone deposition in rabbit cranial defect models. It thus presents a promising avenue for directing stem cell activities and phased biomolecule liberation in skeletal aging renewal.^249^

#### Ultrasound responsive smart scaffolds

Ultrasound technology is regarded as an optimal “remote controller” and “trigger” for bone repair biomaterials. With a high frequency (≥20 kHz), the ultrasound can be concentrated and transmitted in specific media and has applications in clinical areas, including physical therapy and in vivo imaging. Of these, the ultrasound combined with smart scaffolds exhibits the potential to address bone defects as it can promote mechanical stimulation and support vascularization.^250^

**Fig. 9:**
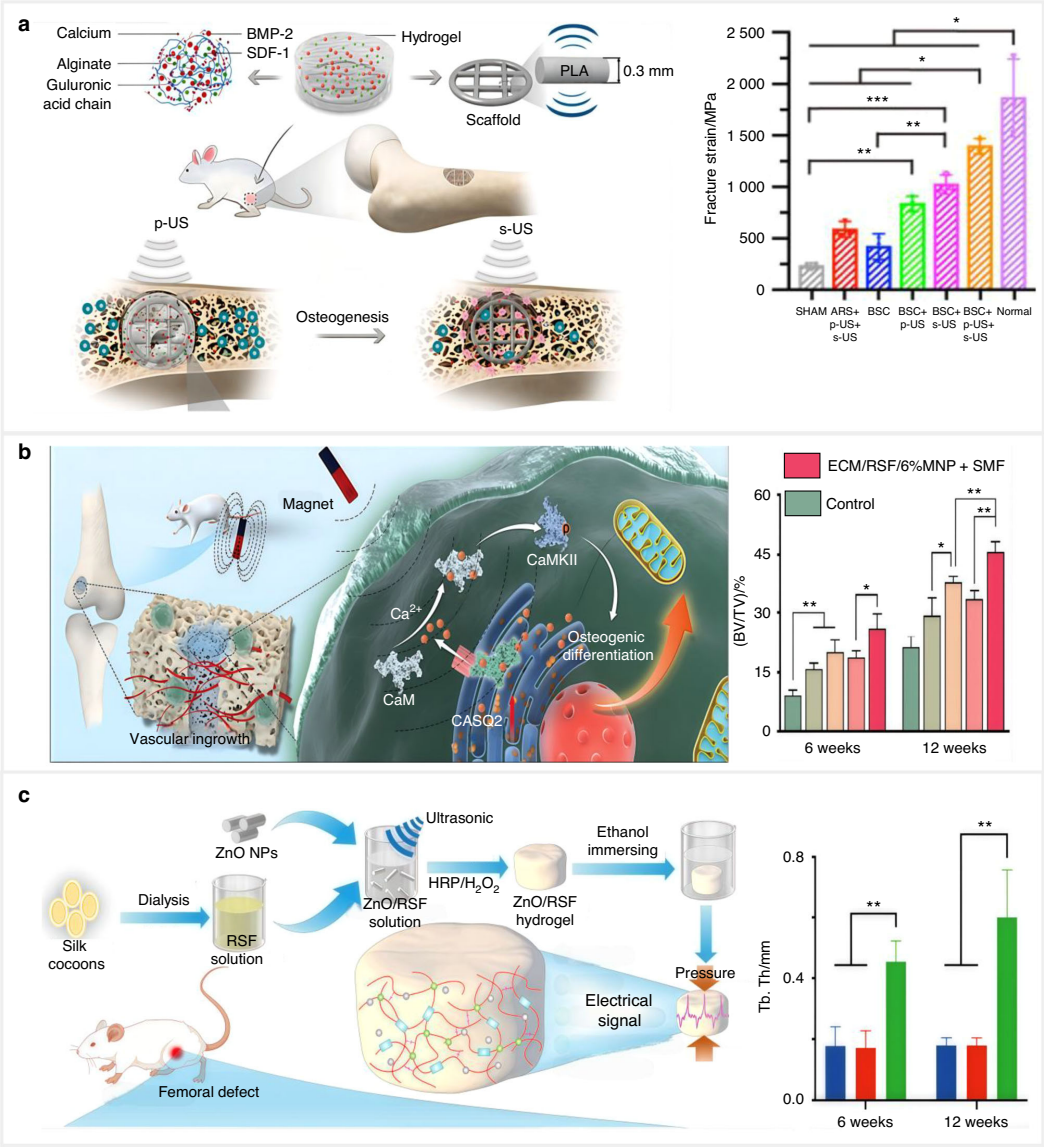
Representative illustration of classic examples of smart scaffolds in response to external stimuli. **a** Schematic of ultrasound-responsive biomimetic hydrogel scaffolds for endogenous BMSC-mediated bone defect repair. Reproduced with permission.^251^ Copyright 2023, KeAi Communications. **b** Illustration of a magnetic-responsive scaffold (ECM/RSF/MNP) for enhanced implant osseointegration. Reproduced with permission.^258^ Copyright 2023, Wiley-VCH. **c** Illustration of zinc oxide (ZnO) nanoparticles and regenerating silk fibroin (RSF) piezoelectric hydrogel facilitating bone regeneration and histomorphometric analysis of bone volume fraction (BV/TV) and trabecular thickness (Tb.Th). Reproduced with permission.^263^ Copyright 2025, Elsevier

Yan et al. developed a bionic hydrogel scaffold complex (BSC), composed of SDF-1/BMP-2 hydrogel and an implanted acoustically responsive scaffold (ARS). Endogenous BMSCs can be recruited to the BSC sites or bone defects by releasing SDF-1/BMP-2 cytokines from the BSC implanted in the damaged bone on demand using pulsed ultrasound (p-US) irradiation with adjusted acoustic parameters. During 14 consecutive days of daily p-US exposure, the alginate hydrogel was deconstructed, causing ARS to be exposed to these aggregated surrounding progenitor cells. The intrinsic resonance of the ARS was evoked by a series of sinusoidal continuous wave ultrasound (s-US) exposures. This produced a highly localized acoustic field on the ARS’s surface and stronger acoustic trapping forces, which ensnared the recruited endogenous stem cells onto the scaffolds and promoted their adhesive growth to achieve in situ bone regeneration (Fig. 9a).^251^ Low-intensity pulsed ultrasound (LIPUS) can also be utilized to activate biomaterials. A scaffold made of decellularized adipose tissue (DAT) and polydopamine-modified black phosphorus nanosheets (pDA-mBP@DAT) has piezoelectric and mild thermogenic properties when exposed to LIPUS. Meanwhile, LIPUS enhances cell attachment, migration, and osteogenic differentiation in this scaffold. After 12 weeks, the pDA-mBP@DAT-LIPUS group showed a bone mineral density of about 770 mg/cm^3^, roughly 5.9 times higher than the control group. This group also had the highest bone volume (BV) of 10.26 mm^3^ ± 1.20 mm^3^ at 12 weeks.^252^

#### Magnetic responsive smart scaffolds

Magnetism has been recognized as a manipulated external stimulation in the bone regeneration process and bone tissue engineering. There are two ways for the scaffolds to be activated by magnetism: direct magnetic forces or external magnetic fields.^253–256^ Incorporating magnetic iron oxide nanoparticles into smart scaffolds is the current manufacturing trend.^257^ The superparamagnetic iron oxide nanoparticles with a diameter of <100 nm contribute to the magnetic responsiveness of the scaffold and inhibit particle aggregation.^247^ These nanoparticles flip the direction of their magnetic torque in response to temperature fluctuations, prohibiting them from retaining their magnetic character after the imposed magnetic field is withdrawn.^247,253^

Tang et al. created a magnetically responsive titanium-based scaffold incorporating cobalt iron oxide (CoFe_2_O_4_) nanoparticles with a P(VDF-TrFE) matrix to enhance osteoblast activity. The low-magnetization layer up-regulated the ALP activity and promoted cell adhesion, proliferation, and differentiation of preosteoblasts. This study made use of magnetism-induced osteogenesis and opened the door to design ideas that make use of an iron oxide covering.^253^ Recently, a biomimetic structural scaffold made of decellularized ECM/regenerated silk fibroin (RSF) and embedded with magnetic nanoparticles (MNP) was fabricated via a simple synthetic approach to emulate the natural architecture and composition of skeleton. The chelated MNP and the β-sheet crystalline of RSF may imitate HAp deposition and increase the scaffold’s compressive modulus by about 20%. An external static magnetic field (SMF) in conjunction with a magnetic-responsive scaffold promotes cell movement, osteogenic development, endotheliocyte neogenesis in vitro, and the bone formation in a critical-size femur-deficient rat model for 12 weeks. Calsequestrin-2-mediated Ca^2+^ release from the endoplasmic reticulum activates the Ca^2+^/calmodulin/calmodulin-dependent kinase II signaling axis, according to RNA sequencing, which explains the molecular underpinnings behind this osteogenic impact (Fig. 9b).^258^

#### Piezoelectric responsive scaffolds

The native piezoelectric behavior of bone attracts extensive attention, which is beneficial for bone repair and remodeling.^259^ Numerous piezoelectric biomaterials similar to the native piezoelectric properties of healthy bone have been fabricated to promote the regeneration of damaged bone.^260^ Peripheral tissue and cells can receive the electrical signal from the biomaterial, which upregulates the signaling pathway to synthesize growth factors.^260^

The composites of piezoelectric biomaterials include ferroelectric (bismuth ferrite, BiFeO_3_), piezoceramics (Barium titanate [BaTiO_3_], zinc oxide [ZnO]), piezoelectric polymers (polyvinylidene fluoride [PVDF], PVDF-TrFE) and polymer/ceramic or glass/ceramic composites.^261,262^ To enhance the bone healing microenvironment via the piezoelectric effect, a self-powered piezoelectric hydrogel was developed by incorporating ZnO nanoparticles into RSF. Adding ZnO to RSF hydrogels not only boosted their mechanical properties by 1.7 times and upped the piezoelectric output by 2.8 times, but it also slowed down the degradation process simultaneously. In vivo studies exhibited appropriate 70% of BV/TV and 0.6 mm trabecular thickness (Tb.Th), which were 2–3 times higher than the control group at 12 weeks, verifying the osteogenic potential of ZnO/RSF piezoelectric hydrogels (Fig. 9c).^263^

#### Electro-responsive smart scaffolds

Electric stimulation has the potential to retain the stemness of BMSCs and promote osteogenesis.^261,264–266^ The essential mechanism is due to the up-regulation of BMPs of BMSCs in response to electrical stimulation, which stimulates the calcium-calmodulin pathway, TGF-β, and other cytokines.^264,265,267^ The direct approach to deliver electroresponsive performance to the scaffold is to incorporate electroactive materials, such as inorganic electroactive materials, metal, graphene, conductive polymers, and carbon nanotubes.^268^ Versatile materials utilizing this strategy to boost osteogenic expression have recently been made. A porous Ti scaffold was made by Zhou et al. and integrated with polypyrrole-polydopamine-HAp (PPy-PDA-HA) film to confer the electroactive property through a layer-by-layer pulse electrodeposition (LBL-PED) method. HA combined with electrical stimulation (300 mV) significantly enhances the differentiation of osteogenic cells on PPy-PDA-HA films. The activated scaffold delivers conductive polymer to stimulate Ca^2+^ influx from activated Ca^2+^ in the channel of the cell membrane, which enhances the Ca^2+^ signal transduction pathway to promote cell adhesion and osteogenesis. Histological images showed that porous scaffolds covered with PPy-PDA-HA produced the most new bone and had a stronger bond with the surrounding host bone tissue.^269^ The potential use of HA as an aged bone regeneration implant is suggested by the differentiation of osteoblasts and bone repair facilitated by the combined effects of HA and electrical stimulation.

### Multifunctional smart scaffolds for skeletal aging

Multiresponsive scaffolds are prudently developed with multiple functionalities, serving as platforms that boost detection sensitivity and drive bone regeneration. As aforementioned, the pathological defect microenvironment in aged bone exhibits high complexity and conveys rich characteristic information, which smart scaffolds can capture to trigger activation.

To enable on-demand activation responsive to the acidic and ROS-rich microenvironment of aged bone, Li et al. created a dual pH/ROS-responsive hydrogel comprising GelMA and hollow MnO_2_ nanoparticles (hMNPs) loaded with BMP-2-associated peptides (hMNP/GelMA). Acid-triggered nanoparticle degradation enabled sustained peptide release over 28 days, while hMNPs catalytically decomposed H_2_O_2_ into H_2_O and O_2_, ameliorating the regenerative microenvironment to enhance osteogenesis. In a skull defect rat model, hMNP/GelMA achieved complete defect coverage with (BV/TV)> 73% at 8 weeks, significantly accelerating the healing process.^270^ Beyond endogenous stimuli, dual exogenous triggers can activate smart scaffolds. Lu et al. constructed a mesoporous bioglass (BG)/chitosan (CS) scaffold (MBCS) functionalized with magnetic SrFe_12_O_19_ nanoparticles that has a thermoregulation synergistic mechanism. The BMP-2/Smad/Runx2 pathway may be activated by the magnetic field produced by MBCS, which could enhance the process of osteogenesis and improve bone growth. The loaded SrFe_12_O_19_ NPs may boost photothermal conversion capacity and raise tumor temperatures to eliminate remaining malignancies when exposed to NIR radiation.^271^

Notably, integrating endogenous and exogenous stimuli has yielded breakthroughs. Fe-CaSiO_3_ composite scaffolds (30CS) with a high compressive strength were recently devised and manufactured by Ma et al. using the 3D printing technique for cortical bone repair and synergistic tumor treatment. The 30CS scaffolds in PBS showed a remarkable photothermal effect, rapidly rising to 50 °C at a power density of 0.6 W/cm^2^ in under 10 min under an 808 nm NIR irradiation. 30CS can release Fe ions to break down H_2_O_2_ in an oxidative environment and offer enough mechanical support for BMSC attachment, growth, and differentiation to improve in vivo bone regeneration.^272^ In addition, an ultrasound-responsive organic hydrogel with piezoelectric property (TBP) was fabricated via PDA-mediated H-bonding of barium titanate (BTO) nanoparticles in tripolyglycerol monostearate gel matrix.^273^ Under LIPUS, TBP uses the piezoelectric property of BTO to produce localized electrical signals. By modulating mitochondrial oxidative phosphorylation via the AKT/GSK3β/β-catenin signaling cascade, TBP enhances the growth, migration, adhesion, and osteogenic differentiation of MC-3 T3-E1 cells. At eight weeks, the in vivo rat skull defect model showed that the bone healing process moved gradually from the fracture border to the middle, suggesting that a conducive healing environment was available for bone development and differentiation. Therefore, these advances highlight the promise of smart scaffolds for regenerating skeletal aging-related defects.

Multiresponsive approaches have a wide range of potential uses outside of bone regeneration, in addition to their extensive use in this field. For instance, using a hard-and-soft integration strategy, Yang et al. created a multifunctional scaffold made of 3D printed microfilaments and a hydrogel network containing aminated ultrasmall superparamagnetic iron oxide nanoparticles (USPIO-NH_2_), simvastatin (SV), and indocyanine green-loaded superamphiphiles. This scaffold enables promoting osteogenic progress and enable non-invasive monitoring of in vivo bone regeneration at the same time.^274^ The biocomposite scaffold demonstrates NIR II fluorescence imaging that is responsive to ALP. Both in vitro and in vivo studies suggested that the scaffold considerably enhances osteogenesis through controlled SV release. The co-crosslinked nanocomposite network’s USPIO-NH_2_ provides a promising approach to the development of theranostic scaffolds for pathological skeletal aging by allowing the detection of scaffold degradation by magnetic resonance imaging.

## Manufactured techniques to fabricate smart materials

Different manufacturing technologies have been utilized in smart materials production to incorporate special characteristics at various scales, which aim to improve the interaction with host tissue in terms of “smartness”. The essential manufacturing method to produce smart materials is the advanced processing method, such as additive manufacturing (AM). With this modification, inert biomaterials can improve their communications with the surrounding environment and attain a certain level of intelligence without functionalization but with the enhancement of bone regeneration. Two types of biomaterials processed techniques, including smart scaffold and smart drug delivery system were discussed. The merits and demerits are listed in Table 2.

**Table 2:** The advantages and limitations of smart materials manufacturing techniques

	Type	Techniques	Advantages	Limitations	Ref.
Smart drug delivery system	Polymeric-based nanocarriers	Self-assembly	Simplicity and efficiency, scalability	Variability in structure, unpredictable release profiles	^277^
		Nano-precipitation	Speed, simplicity, and the capacity to form a wide variety of architectures	Reduced particle stability, reduced drug loading efficiency, and drug leakage	^338^
		Templated assembly	Tunable and robust, forms hybrid nanoparticles, and utilizes a diverse array of building blocks	Complex and multistep, requires careful reactant choice, expensive	^339^
	Liposome based nanocarriers	Thin film hydration	Easy-to-operate, controlled size and charge, cost-effectiveness	Heterogeneous production, low entrapment capacity, difficulty in removing organic solvents and scale-up	^340^
		Solvent injection	Simple and rapid process, tailorable size and composition	Solvent removal required, potential contamination, unpredictable release kinetics	^341^
		Supercritical fluid method	Cheap and environmentally harmless solvent, control of particle size, in situ sterilization, and the possibility of large-scale production	High equipment costs, limited availability of supercritical fluids	^284^
	Ceramic-based nanocarriers	Sol-gel approach	Precise control over the composition and porosity, easily scalable	Excessive cost, moisture sensitivity, and brittle issues	^342^
		Co-precipitation	High drug loading capacity, control over particle size and morphology	Complexity of process control, risk of aggregation, limited control over surface properties	^343^
	Metal-based nanocarriers	Top-down strategies	Precise control over size and shape, potential for multifunctionality	Complexity of processes, material waste	^344^
		Bottom-up strategies	High surface area-to-volume ratio, scalability	High setup costs, limited material compatibility, thermal sensitivity	^344^
Smart scaffolds	Metal scaffolds	Electron beam melting (EBM)	Rapid prototyping and production, excellent corrosion resistance, and osteogenesis	Rough surface, poor integrity, and dimensional errors, post-processing needs	^345^
		Selective laser melting (SLM)	Excellent mechanical properties, superior dimensional precision, and customization	High level of surface roughness, post-processing requirements	^346^
		Selective laser sintering (SLS)	Functionally graded porosity, rapid prototyping	Thermal management issues, and technical expertise required	^347^
	Bioceramic scaffolds	Binder jetting (BJT)	Free of support structure, a low amount of sacrificial material, and large-scale production	Need for sintering, issue of brittleness, residual binder issues	^348^
		Digital light processing (DLP)	Higher precision, smooth surfaces, shorter manufacturing time, lower cost	Requires a manual post-processing step, high equipment, and material costs	^349^
		Sheet lamination (SHL)	High throughput, desirable density characteristics, simple process	Post-processing requirement, limited design flexibility	^350^
	Polymer scaffolds	Fused deposition modeling (FDM)	Cost-effectiveness and rapid prototyping	Weaker structures, specific material needs, post-processing requirements	^351^
		Selective laser sintering (SLS)	Customization and excellent mechanical properties	Complex process control, thermal management issues	^352^

### Fabrication of smart drug delivery systems

Nanoparticle drug delivery has received widespread attention due to its advantages of bioavailability improvement and precise drug delivery.^275^ They can be divided into four main groups based on their physical properties (e.g., morphology and size) and chemical characteristics: polymers, liposomes, ceramics, and metal nanocarriers.^276^ A variety of techniques can be used to generate the desirable nanocarriers with regulated size, shape, stability, functionality, and drug-loading efficiency.

#### Polymeric-based nanocarriers

The commonly used methods for polymeric nanoparticle production include self-assembly, nanoprecipitation, and templated assembly (Fig. S1). Self-assembly refers to the process of spontaneous arrangement of different polymer strands into a highly controlled suspension of particles and is, to date, the most famous method for fabricating polymer nanoparticles.^277^ The spontaneous process relies on intrinsic energy from the system, including hydrogen bonding, covalent bonding, repulsive forces, and supramolecular attraction to achieve the entropic equilibrium of the system.^278^ Nanoprecipitation is another easy-to-operate method that does not require additional forced energy and can be used to fabricate polymer nanoparticles for a wide range of payloads. It depends on the mechanism of self-assembly of preformed polymers into nanoparticles and requires both aqueous and organic phases.^279^ The fabricating process needs to be controlled since the characteristics could be changed by various factors, such as polymer nature, concentration, and injection method.^280^ Template assembly uses a template as a substrate coated with a polymer ingredient. Templates include inorganic nanoparticles and even cell models, whose properties can alter the characteristics of the nanoparticles. Depending on the template selection, degradable templates, such as pH-responsive calcium carbonate nanocarriers, can be used to create polymeric nanocarriers.^281^

#### Liposome-based nanocarriers

Thin film hydration, solvent injection techniques, and supercritical fluid methods are the commonly employed techniques for liposome production (Fig. S2). Simple thin film hydration is the first reported method used for liposome-based nanocarriers with various architectural arrangements.^282^ Also, liposomes can be prepared by the solvent injection technique. In this method, liposomes are formed by rapidly injecting lipids dissolved in an organic solvent (ethanol or ether) into an aqueous medium.^283^ Based on the conventional method, the novel process represented by supercritical fluid addressed problems of structural control and scale-up. It employs a supercritical fluid, such as carbon dioxide (CO_2_), that is maintained under supercritical conditions (temperature and pressure) to produce liposomes.^284^

#### Ceramic-based nanocarriers

The well-designed fabrication methods allow for the excellent mechanical and ideal elastic modulus of nanoceramics. Common fabrication strategies involve the sol-gel technique and co-precipitation (Fig. S3).^285^ The sol-gel technique could form a complex structure (e.g., metal oxide and ceramic nanopowders) from chemically homogeneous gel phases that do not need high processing temperatures. The powder of alumina/ZrO_2_ composite could be generated by the co-precipitation method through the process of mixing, precipitation, filtering, washing, and drying.

#### Metal-based nanocarriers

The production of metallic nanoparticles includes three different approaches physical, chemical, and biological methods. The physical method refers to the top-down strategies, while the last two methods are referred to as bottom-up strategies (Fig. S4).^286^ Within the top-down approach, the nanoparticles are created by a volume reduction method in which large pieces of material are broken down into smaller pieces of material. This can be achieved by using an ultrasonic generator with a frequency of about 1.2 MHz and a high intensity. The commonly utilized top-down methods include mechanical milling, electrospinning, laser ablation, sputtering, electron explosion, sonication, pulsed wire discharge method, arc discharge method, and lithography.^287^ In the bottom-up assembly process, nanostructures are created particle by particle or atom by atom. It can be achieved by the growth of atomic nuclei after an elevated level of supersaturation. Commonly, bottom-up methods for metal-based nanocarrier fabrication are composed of chemical vapor deposition, sol-gel process, co-precipitation, molecular condensation, hydrothermal, and biological synthesis.^288^

#### Fabrication of smart scaffolds

The combined advantageous properties of smart scaffolds come from the elaborated and sophisticated microstructure, which requires precise control of the manufacturing process. Conventional techniques, including solvent casting, particulate leaching, and gas foaming, are not sufficient to regulate the manufacturing process and realize the beneficial properties of smart scaffolds.^289^ AM (3D printing) is developed to produce patient-tailored scaffolds with interconnected porous structures that are prone to angiogenesis and osteogenesis.^166,290,291^ Herein, the respective AM methods related to smart scaffolds with different performances, including metal, ceramic, and polymer scaffolds, have been discussed.

#### AM for metal scaffolds

Laser powder bed melting is a commonly employed approach to fabricate metal scaffolds with more process precision and surface performance, including electron beam melting (EBM), selective laser melting (SLM), and selective laser sintering (SLS) (Fig. S5). EBM refers to the powder bed fusion techniques (PBF) that solidify particles with high-speed electron beams layer by layer in a vacuum. Pure CoCr, Ti, and Ti alloys-based scaffolds have been processed by EBM, presenting good corrosion resistance and osteogenesis.^292,293^ SLM defines the PBF that melts and solidifies particles with a laser source layer by layer surrounded by inert gas protection. Pure Ta and Ti-TaNb-Zr alloy-based scaffolds have been produced by the SLM techniques.^294^ Although with the risk of oxidation, SLM has been utilized to fabricate the Mg- and Zn-based biodegradable scaffold.^295^ SLS is recognized as the ideal method to produce a function-graded porous scaffold to mimic the natural anatomical structure. Pure metal or polymer composite biomaterial, such as PLLA/Mg-based scaffold, could be fabricated by this method.^296^ A diversity of composite scaffolds could be synthesized by this approach.

#### AM for bioceramic scaffolds

Traditional methods, such as casting and sintering, are expensive and time-consuming and cannot meet the high demand of ceramics production. Therefore, faster and more convenient additive manufacturers, such as powder-based, slurry-based, and bulk solid-based approaches, are being developed to fabricate sophisticated bioceramic scaffolds (Fig. S6). Powder-based deposition techniques like binder jetting (BJT) cement the ceramic particles from the powder bed layer by layer, via diffusion of a liquid binder or by thermal energy provided by a laser beam. Using this technique, various ceramics, including HAp, TCP, and CS have been produced as bone scaffolds.^297^ Slurry-based AM includes liquid or semi-liquid systems with various viscosities and solids content, which are used to dissolve the ceramic particles. Of these, digital light processing (DLP) has received more attention in bone engineering for inks applied to produce ceramic scaffolds. DLP technology enables small products with complex geometries to be printed with high precision using liquid photopolymers as the structuring material. It requires a manual post-processing step and polymerization shrinkage can affect the printed part.^298^ Bulk solid-based AM typically includes sheet lamination (SHL) to construct ceramic scaffolds. The SHL utilizes subtractive and additive strategies to create the parts by connecting layer after layer of solid sheets through heat and pressure and thermally bonded coatings. Various ceramic products have been generated by this method, including aluminum nitride, SiC composites, and monolithic silicon carbide.^299^

#### AM for polymer scaffolds

Compared to conventional methods, such as electrospinning and gas molding, AM produces polymer scaffolds with ideal porosity, tailored structures, and repeatable procedures. The commonly employed 3D printing techniques, including fused deposition modeling (FDM) and selective laser sintering (SLS), can be utilized to fabricate polymer-based scaffolds (Fig. S7). Several composite scaffolds that combine ceramic and polymers can be produced by FDM, such as PLA with HAP or β-TCP.^300^ SLS is another AM technique that is associated with polymer materials, using the powder form of particles to construct the polymer parts of the scaffold. Various types of polymers including PCL, PLLA, PVA, and PLGA could be fabricated by SLS.^289^

## Clinical translations: challenges and future directions

New advancements in fundamental studies are providing a superior understanding of the mechanisms of many disorders and physiological events. There are numerous novel biomedical techniques and products stemming from the academic world.^301^ However, it has been difficult to translate this biomedical technology into the clinic. Less than 5% of life science research findings that originate in academia are transformed into novel drugs, equipment, diagnostics, or enhancements to the clinical setting. Approximately 90% of “translational” research initiatives in academia are never tested in humans.^302,303^ There are substantial differences between the number of biomedical advances developed by scholars and what is offered to clinicians and patients.

Several acknowledged obstacles exist that prevent the application of fundamental scientific understanding to practical ends. Inadequate in-depth mechanistic research, absence of standardized animal models, misaligned academic evaluation systems, disconnect from clinical contexts, and lack of translational medicine platforms are all examples of academic obstacles.^85^ Overlooking the biomaterials-host immune interaction and the presence of age-related biological variables makes most studies statistically significant, but not clinically significant results. Immune rejection of foreign biomaterials has been a long-standing challenge that has led to the short lifespan of the products. It also affects the immune balance of the host, which makes them susceptible to allergy or infection.^304^ Sicari et al. demonstrated significant age-dependent variations in the composition and regenerative remodeling properties of porcine small intestinal submucosa extracellular matrix (SIS-ECM) bioscaffolds. Specifically, SIS harvested from donor pigs aged 3, 12, 26, and >52 weeks exhibited distinct functional differences.^305^ Industrial system confronts challenges such as complicated large-scale production processes, stringent quality control demands, high upfront research and development expenses, and uncertain market reception.^306^ The ability to scale up proof-of-concept studies from the laboratory to industrial-scale automated production with safety, efficiency, uniformity, reproducibility, and acceptable cost is a practical challenge for all clinical translation facilities. Regulatory issue is always a major concern for the governmental sector, including blurred boundaries with diverse classification, failure to promptly update evaluation criteria and computational techniques for physical tests, absence of innovative assessment frameworks, complexity and cumbersomeness of approval procedures for market entry, insufficient allocation of financial resources, and limitations of incentive-based policies.^307^ The process of converting preclinical academic research into clinical application is exceptionally difficult, costly, and time-consuming. Consequently, several biomaterial solutions that exhibit evident effectiveness and safety in preclinical settings are unable to make it to market.^308^

Although there have been no clinical reports on the use of smart biomaterials in human bone tissue to date, relevant personnel in academia, government, and industry have made considerable efforts to promote the clinical transformation of smart materials.^309–312^ Several bioactive and biodegradable materials have been approved for clinical use.^313^ For example, iFactor is a substance that combines P-15 with bone minerals and is suspended in a hydrogel. In November 2015, the FDA authorized it for the reconstruction of deteriorated cervical discs at one level (C3-C4 to C6-C7) following a single-level discectomy for intractable radiculopathy. iFactor was used to treat symptomatic radiculopathy caused by single-level cervical degenerative disc degeneration. At the 2-year mark, a randomized single-blind research with 319 patients showed that it was safe and effective. Most of the patients (97.30%, 144/148) had fusion success.^314^ Recently, the world’s first approved magnesium-containing biodegradable polymer bone repair material (Approval Number: 20253130952), developed by Qin Ling et al. over 15 years, has received Class III implant approval as an Innovative Medical Device from China’s National Medical Products Administration (NMPA). The launch of these products has provided valuable experience for future research and development and clinical translation of smart biomaterials. First, academia should foster an environment conducive to the early stages of translation by determining clinical needs, assembling interdisciplinary teams, reforming academic promotion systems, collaborating with technology transfer offices and establishing regional translation centers. A deep understanding of clinical needs through communication with clinicians is essential to guide material design, performance, and fabrication. Second, industry effort can be shown through collaborations, financial incentives, and support for mentorship and education programs. The systematic analysis of competing products based on factors such as necessity, comparative advantages, ease of operation, and cost-income ratio significantly impacts commercial success.^313^ Standardized procedures for the production, evaluation, and approval of AM-based biomaterials can expedite the procedure and make it easier for clinical practice to use them. Third, regulatory departments’ leadership can be demonstrated by reducing excessive government control and offering flexibility in mandatory preclinical organoids and animal research.^315^ From the start of the creation of smart biomaterials, strong collaboration between the scientific community, physicians, engineers, industry players, regulatory agencies, patients, and patient advocacy groups is essential as the area develops.

Furthermore, three emerging avenues for smart biomaterial development that we anticipate will solve current issues and create fresh chances in precise medicine. Continuously emerging biomaterial innovations may be expected to keep these opportunities forward. Integrating artificial intelligence (AI) and machine learning/deep learning algorithms can improve scaffold design, improve target efficiency, balance immune interaction and further personalize treatment plans.^316^ By leveraging patient clinical and genetic data, researchers can now predict how materials will behave in biological systems, optimize the design of scaffolds, enhance benefits while minimizing side effects and accelerate the development of novel biomaterials with tailored properties.^317^ With AI-guided customized medicine, researchers can improve the safety and effectiveness profiles of smart biomaterials while cutting down on the time and expense involved in their design and production.

Real-time in vivo sensing of responsive biomaterials refers to the ability to monitor and react to the pathological environment of aged bone, and specifically, to feed back into the healing process of neo-bone tissue. Yang et al. combined highly selective ALP probes (LET-3) with PCL/CS composite scaffolds to create a novel kind of “therapeutic self-monitoring scaffold” for bone regeneration. In addition to stimulating osteoblast proliferation and differentiation, the LET-3 assigned scaffolds made it possible to utilize NIR-FL/PA dual-mode imaging for non-invasive, high-resolution, immediate examination and semi-quantitative quantification of ALP expression both in vitro and in vivo.^318^ The real-time in vivo bone-sensing smart biomaterials can dynamically adjust treatment protocols based on the actual status of bone tissue, enhance therapeutic outcomes and promotes the swift recovery of bone tissue.

Combining with real-time sensing systems, the closed-loop strategies offer advanced therapeutic potential through smart feedback disease management. Jiang et al. created a flexible bioelectronic system composed of wirelessly powered, closed-loop sensing and stimulation circuits with skin-interfacing hydrogel electrodes. It can adhere and detach on demand in order to offer programmed electrical cues for rapid wound healing. By continually monitoring skin temperature and impedance, the wound care system can provide electrical stimulation based on the wound environment, resulting in approximately 25% faster healing and a 50% improvement in dermal remodeling as compared to control.^319^ Closed-loop therapeutic systems deliver dynamic, personalized, and precise treatment via a “sense-respond-adapt” feedback loop. They drive biomaterials to transition from static carriers to dynamic therapeutic entities, which act as an example for smart biomaterials for aged bone repair and regeneration.

## Conclusion

Smart biomaterials, such as smart scaffolds and drug release systems, offer superior capacity to stimulate regeneration and repair of aged bones than traditional treatments. We provided an overview of a range of intelligent signal responses for the systemic smart drug release system, including endogenous (e.g., enzymes, pH, temperature, specific ions, ROS and immune) and exogenous (e.g., light, magnetism, ultrasound, and mechanical force) stimuli. When stimuli are applied, smart drug carriers encounter phase transitions and conformational changes that release bioactive components in a site-specific, time-release and dose-controllable manner. In terms of smart scaffolds, we have reviewed some special features and designs of smart stimulus-responsive scaffolds for skeletal regeneration in the elderly. Intelligent scaffolds imitate the structure and biological components of healthy bones. In addition to providing physicochemical and physical signals to support integration with cells and initiate bone healing, they can deliver drugs and bioactive substances in response to the threshold stimuli. Beyond limited single stimulation dependency, the construction of multi-stimulation and multifunction smart biomaterials could increase the accuracy of medication delivery and exhibit synergistic effects for skeletal aging repair and regeneration. We have also presented advanced technologies for the fabrication of smart biomaterials and proposed strategies and challenges for future clinical translation of smart biomaterials, emphasizing AI-driven therapeutic frameworks for personalized biomaterial design. Smart biomaterials for aged bone repair and regeneration will demonstrate significant promise and benefits with further development and precise clinical translation.

## Supplementary information

Supplementary materials
