# Supplementary material for: Smart biomaterials for skeletal aging repair and regeneration
Source: Bone Res. 2026 Feb 14;14:24. doi: 10.1038/s41413-026-00505-9 (PMC12905451; doi:10.1038/s41413-026-00505-9)
Supplement: Supplementary file 1 — Supplementary materials [file 41413_2026_505_MOESM1_ESM.docx]

**Supplementary materials**

**Smart biomaterials for** **skeletal aging repair and** **regeneration**

**Running title:** Smart materials for skeletal aging repair

**Authors**

Dingfa Liang^1,2,3^, Hufei Wang^1,2^, Yu Jiang^1,2,3^, Zeyuan Zhang^1,2,3^, Tianjunke Zhou^1,2,3^, Siliang Ge^1,2,3^, Shuhuai Tan^1,2,3^, Kaihua Qin^1,2,3^, Yilin Wang ^1,2,3^, Xisheng Lin^3,4^, Yong Xie^1,2*^, Houchen Lyu^1,2*^, Licheng Zhang^1,2*^

**Affiliations**

1. Department of Orthopedics, The Fourth Medical Center of Chinese PLA General Hospital, Beijing, 100048, China
2. National Clinical Research Center for Orthopedics, Sports Medicine and Rehabilitation, Beijing, China.
3. Medical School of Chinese PLA, Beijing, 100853, China.
4. Department of Rehabilitation, the Second Medical Center of Chinese PLA General Hospital, Beijing, 100853, China.

**Correspondence Author**

Yong Xie*

Department of Orthopedics, Chinese PLA General Hospital, No. 28, Fuxing Road, Beijing, 100853, People’s Republic of China.

Email: yong.xie301@outlook.com

Houchen Lyu*

Department of Orthopedics, Chinese PLA General Hospital, No. 28, Fuxing Road, Beijing, 100853, People’s Republic of China.

Email: houchenlyu@301hospital.com.cn

Licheng Zhang*

Department of Orthopedics, Chinese PLA General Hospital, No. 28, Fuxing Road,

Beijing, 100853, People’s Republic of China.

1. mail: [zhanglcheng218@126.com](mailto:zhanglcheng218@126.com)

**Word counts**

Abstract: 242

Main text: 16,172

**Author Contributions**

D.L. and H.W. contributed equally to this work. Conceptualization and design: L.Z., H.L., Y.X. and D.L.; writing-original draft preparation: D.L.; writing-review and editing: D.L., H.W., Y.J. and Z.Y.; revision for intellectual content: all authors.


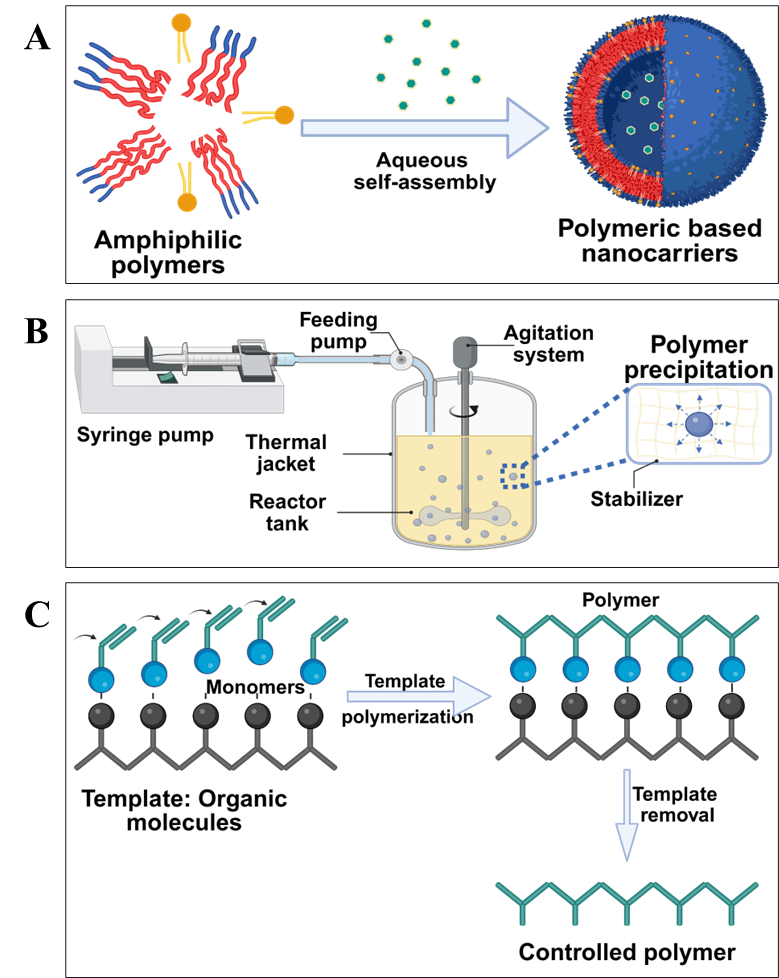


**Fig. S1:** Schematic flowchart illustrating the preparation of polymeric nanocarriers. A: Self-assembly method. B: Nano-precipitation technique. C: Templated assembly approach.


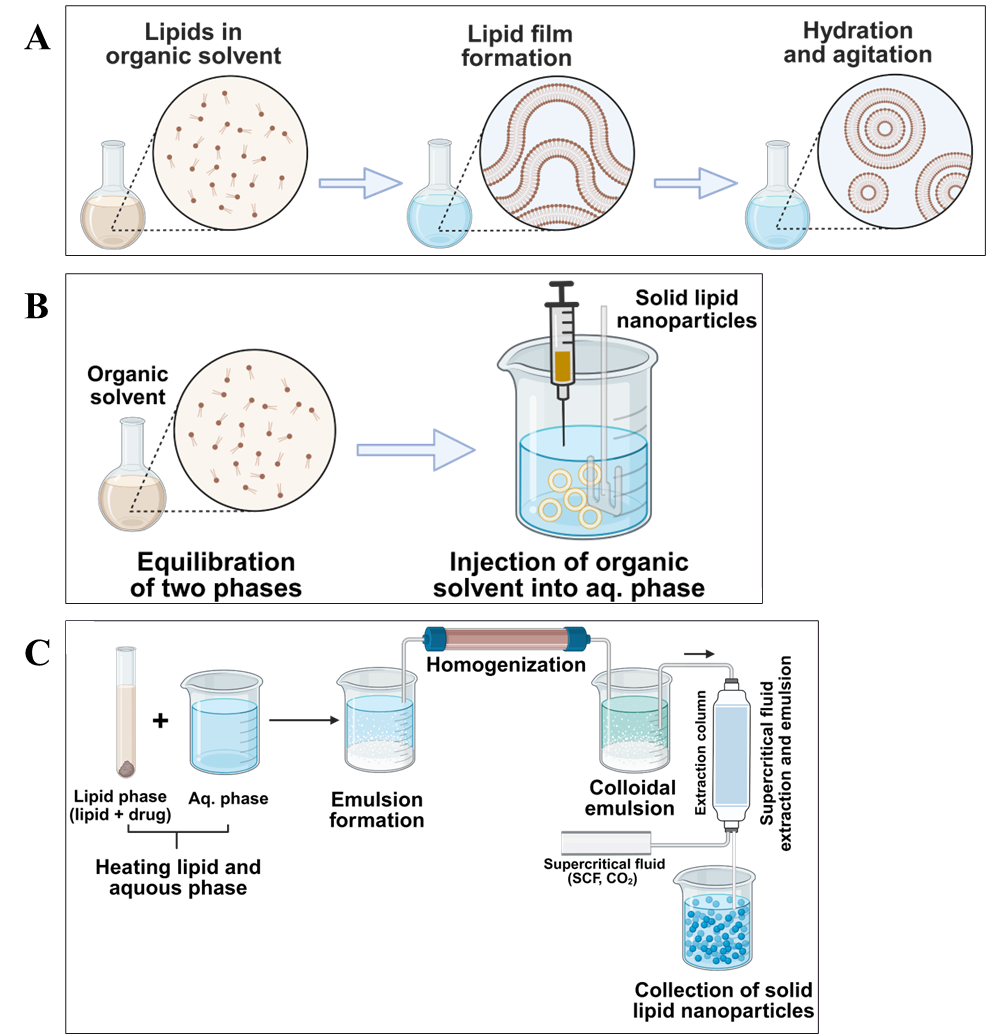
 **Fig. S2:** Schematic diagram of the fabrication process for liposomal nanocarriers. A: Thin film hydration. B: Solvent injection. C: Supercritical fluid method.


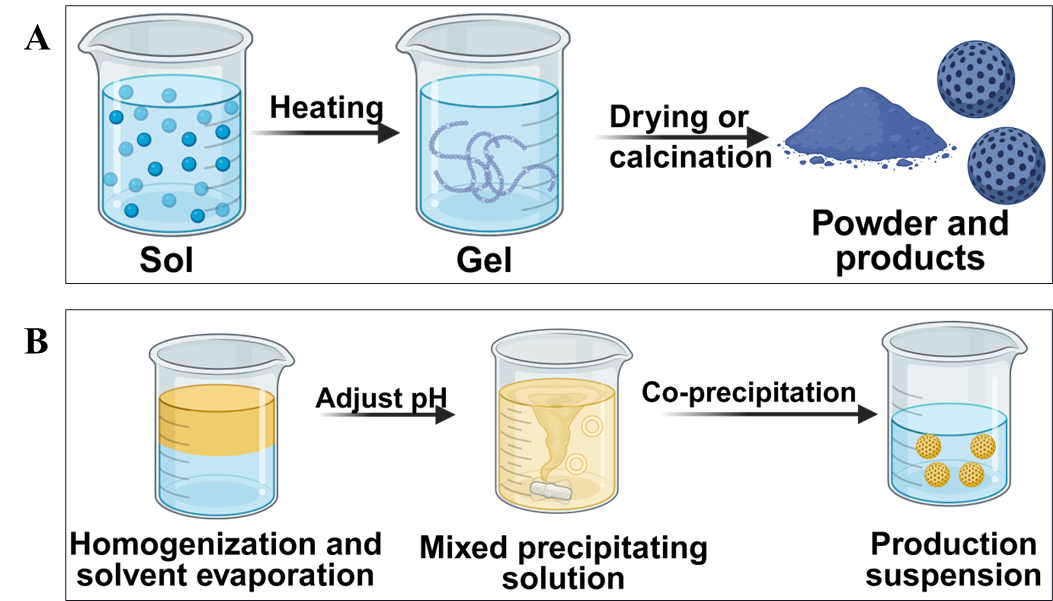


**Fig. S3:** Flowchart depicting the synthesis of ceramic-based nanocarriers. A: Sol-gel method. B: Co-precipitation process.


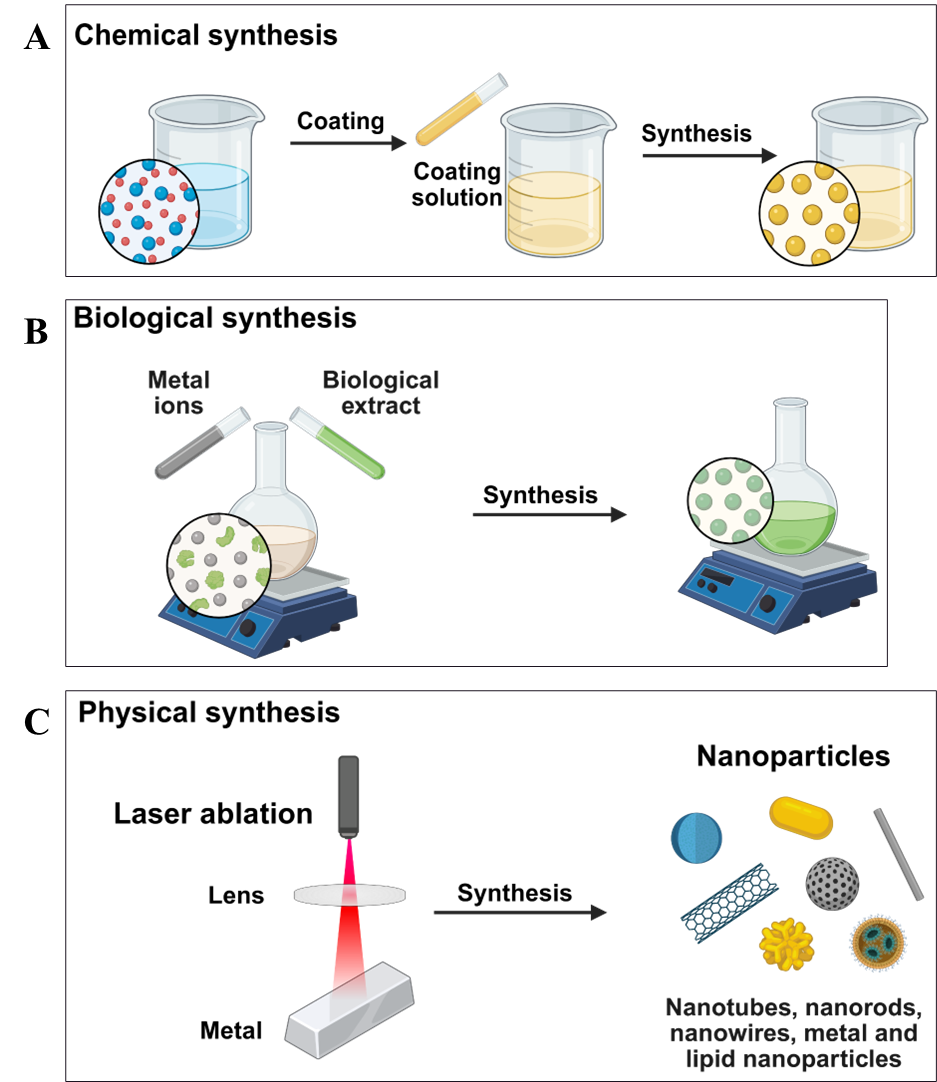


**Fig. S4:** Schematic representation of the preparation routes for metal-based nanocarriers. A: Chemical synthesis. B: Biological synthesis. C: Physical synthesis.


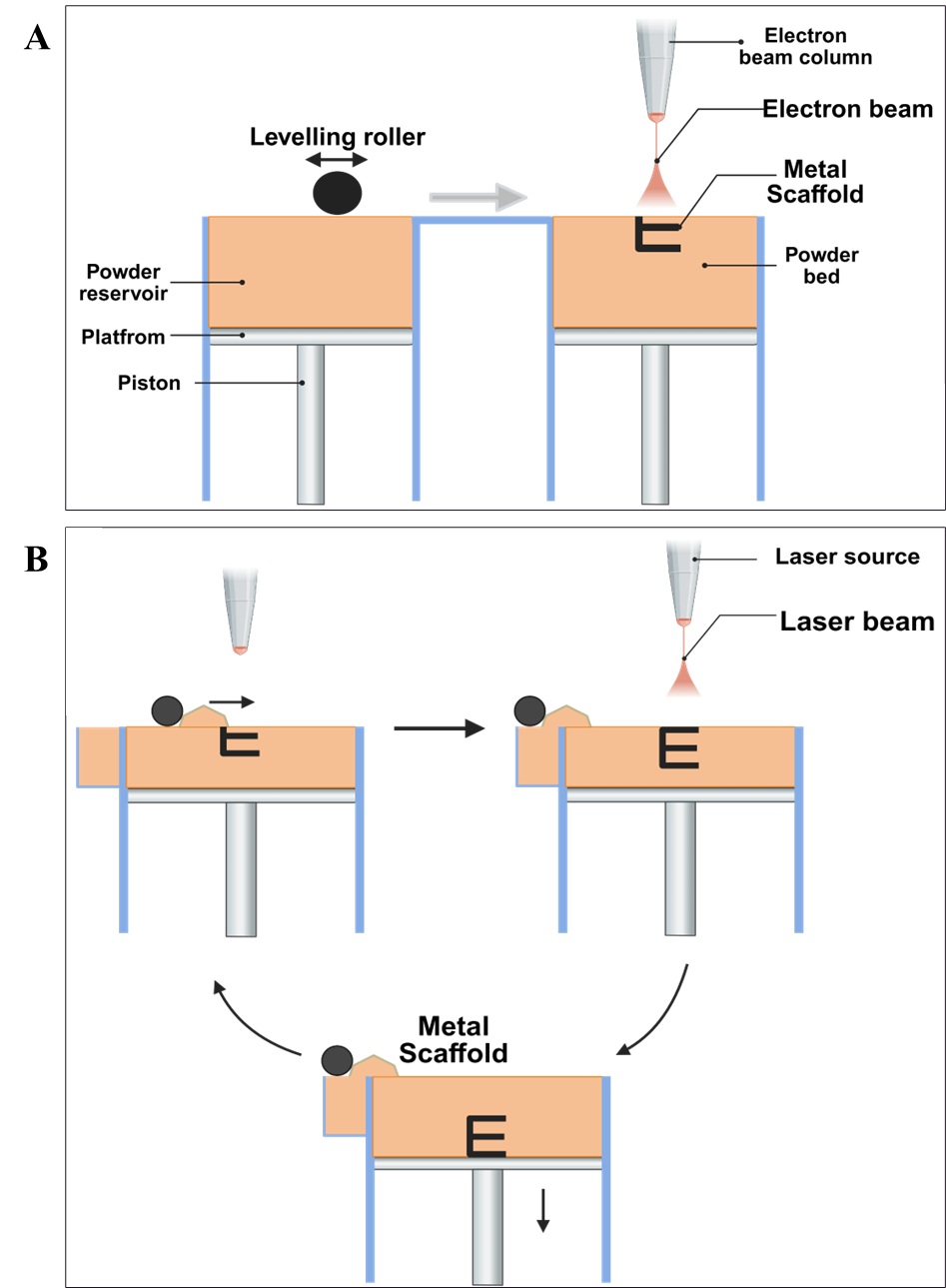


**Fig. S5:** Fabrication flowchart for metallic scaffolds. A: Electron beam melting (EBM). B: Selective laser melting (SLM) and selective laser sintering (SLS).


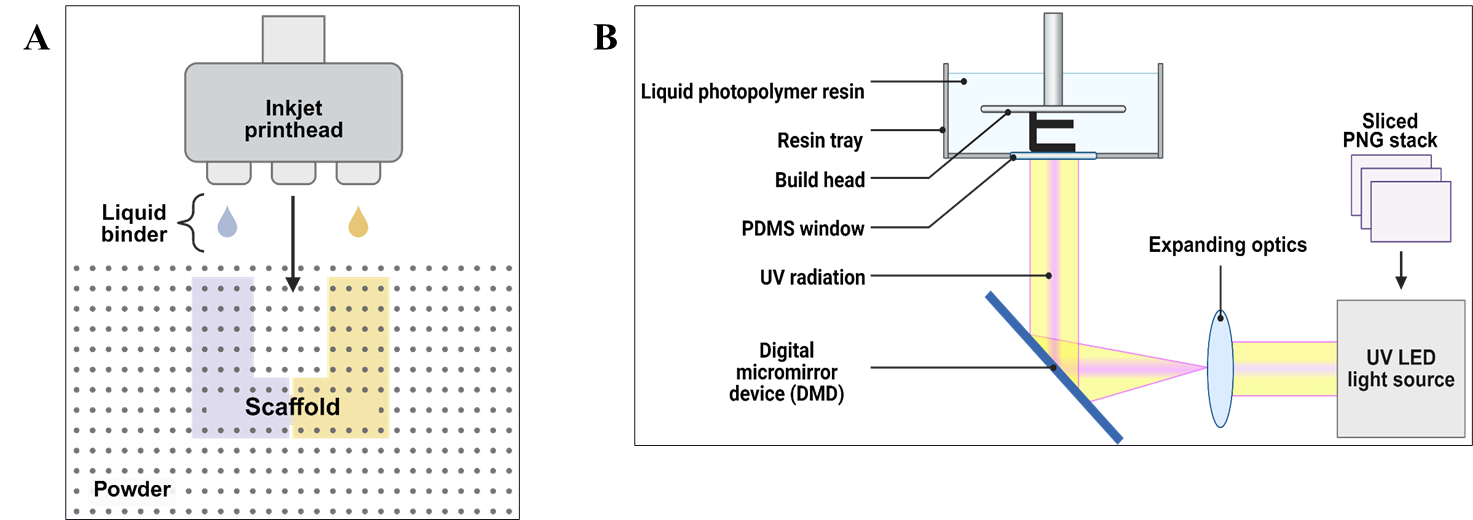


**Fig. S6:** Schematic flowchart of the manufacturing process for bioceramic scaffolds. A: Binder jetting (BJT). B: Digital light processing (DLP).


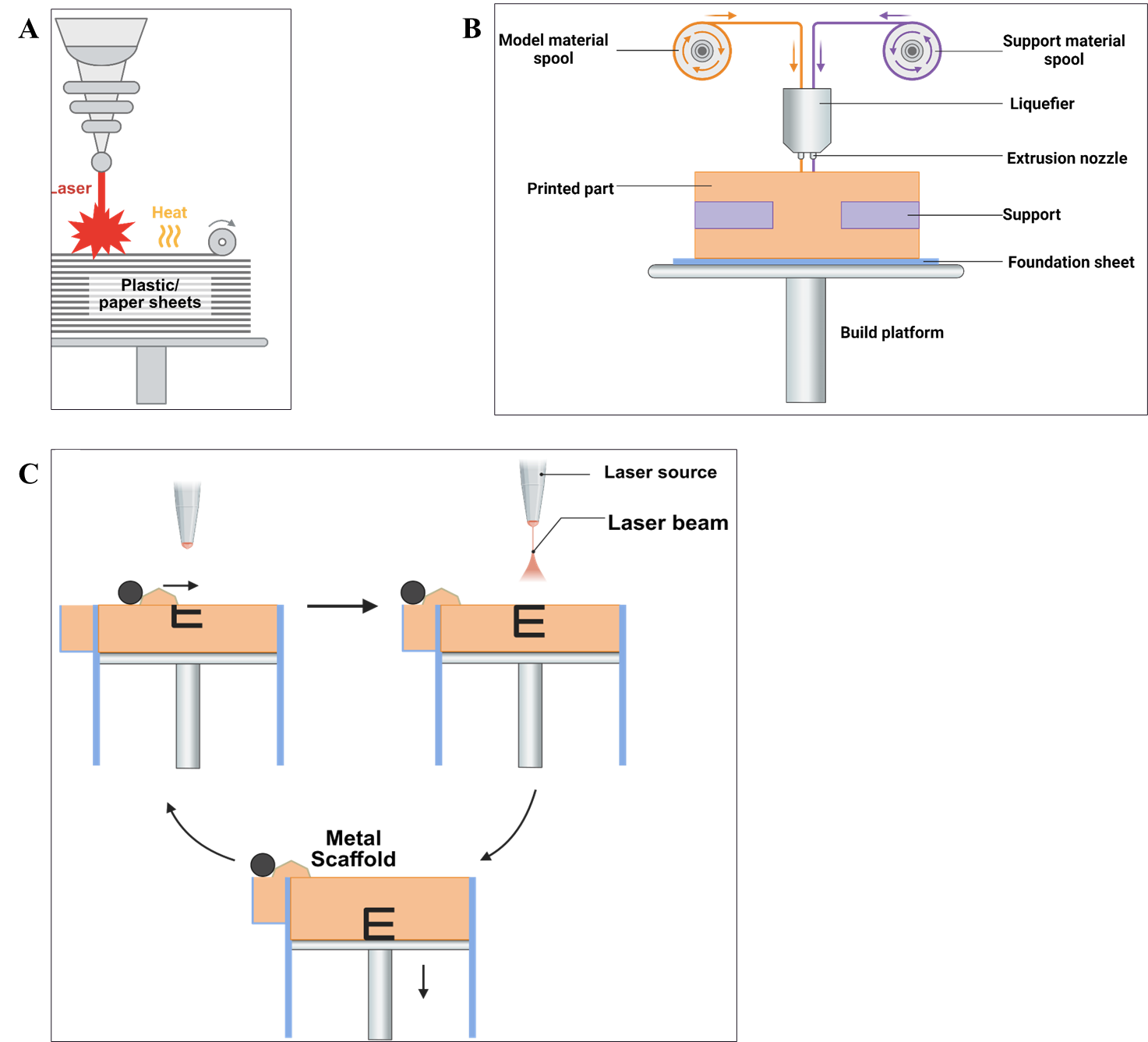


**Fig. S7:** Process flow diagram for polymer scaffold fabrication. A: Sheet lamination (SHL). B: Fused deposition modeling (FDM). C: Selective laser sintering (SLS).
